# Neurofilament proteins as a potential biomarker in chemotherapy-induced polyneuropathy

**DOI:** 10.1172/jci.insight.154395

**Published:** 2022-03-22

**Authors:** Petra Huehnchen, Christian Schinke, Nikola Bangemann, Adam D. Dordevic, Johannes Kern, Smilla K. Maierhof, Lois Hew, Luca Nolte, Peter Körtvelyessy, Jens C. Göpfert, Klemens Ruprecht, Christopher J. Somps, Jens-Uwe Blohmer, Jalid Sehouli, Matthias Endres, Wolfgang Boehmerle

**Affiliations:** 1 Charité – Universitätsmedizin Berlin, corporate member of Freie Universität Berlin and Humboldt Universität zu Berlin, Klinik und Hochschulambulanz für Neurologie, Berlin, Germany.; 2 Charité – Universitätsmedizin Berlin, corporate member of Freie Universität Berlin and Humboldt Universität zu Berlin, NeuroCure Cluster of Excellence, Berlin, Germany.; 3 Berlin Institute of Health at Charité — Universitätsmedizin Berlin, Berlin, Germany.; 4 Carl-Thiem Clinic Cottbus, Clinic for Senology and Systemic Gynecological Oncology, Breast Cancer Center, Cottbus, Brandenburg, Germany.; 5 Charité – Universitätsmedizin Berlin, corporate member of Freie Universität Berlin and Humboldt-Universität zu Berlin, Einstein Center for Neurosciences Berlin, Germany.; 6 German Center for Neurodegenerative Diseases, Magdeburg, Sachsen-Anhalt, Germany.; 7 Natural and Medical Sciences Institute (NMI) at the University of Tübingen, Reutlingen, Baden-Württemberg, Germany.; 8 Drug Safety Research and Development, Pfizer, Groton, Connecticut, USA.; 9 Charité – Universitätsmedizin Berlin, corporate member of Freie Universität Berlin and Humboldt Universität zu Berlin, Klinik für Gynäkologie mit Brustkrebszentrum, Berlin, Germany.; 10 Charité – Universitätsmedizin Berlin, corporate member of Freie Universität Berlin and Humboldt Universität zu Berlin, Klinik für Gynäkologie mit Zentrum für onkologische Chirurgie, Berlin, Germany.; 11 Charité – Universitätsmedizin Berlin, corporate member of Freie Universität Berlin and Humboldt Universität zu Berlin, Center for Stroke Research Berlin, Berlin, Germany.; 12 German Center for Neurodegenerative Diseases, Berlin, Germany.; 13 German Center for Cardiovascular Research (DZHK), Partner Site Berlin, Berlin, Germany.

**Keywords:** Neuroscience, Adult stem cells, Cancer, Toxicology

## Abstract

**BACKGROUND:**

Paclitaxel chemotherapy frequently induces dose-limiting sensory axonal polyneuropathy. Given that sensory symptoms are challenging to assess objectively in clinical practice, an easily accessible biomarker for chemotherapy-induced polyneuropathy (CIPN) holds the potential to improve early diagnosis. Here, we describe neurofilament light chain (NFL), a marker for neuroaxonal damage, as a translational surrogate marker for CIPN.

**METHODS:**

NFL concentrations were measured in an in vitro model of CIPN, exposing induced pluripotent stem cell–derived sensory neurons (iPSC-DSNs) to paclitaxel. Patients with breast or ovarian cancer undergoing paclitaxel chemotherapy, breast cancer control patients without chemotherapy, and healthy controls were recruited in a cohort study and examined before chemotherapy (V1) and after 28 weeks (V2, after chemotherapy). CIPN was assessed by the validated Total Neuropathy Score reduced (TNSr), which combines patient-reported symptoms with data from clinical examinations. Serum NFL (NFL_s_) concentrations were measured at both visits with single-molecule array technology.

**RESULTS:**

NFL was released from iPSC-DSNs upon paclitaxel incubation in a dose- and time-dependent manner and was inversely correlated with iPSC-DSN viability. NFL_s_ strongly increased in paclitaxel-treated patients with CIPN, but not in patients receiving chemotherapy without CIPN or controls, resulting in an 86% sensitivity and 87% specificity. An NFL_s_ increase of +36 pg/mL from baseline was associated with a predicted CIPN probability of more than 0.5.

**CONCLUSION:**

NFL_s_ was correlated with CIPN development and severity, which may guide neurotoxic chemotherapy in the future.

**TRIAL REGISTRATION:**

ClinicalTrials.gov NCT02753036.

**FUNDING:**

Deutsche Forschungsgemeinschaft (EXC 257 NeuroCure), BMBF (Center for Stroke Research Berlin, 01 EO 0801), Animalfree Research, EU Horizon 2020 Innovative Medicines Initiative 2 Joint Undertaking (TransBioLine, 821283), Charité 3R — Replace — Reduce — Refine.

## Graphical abstract

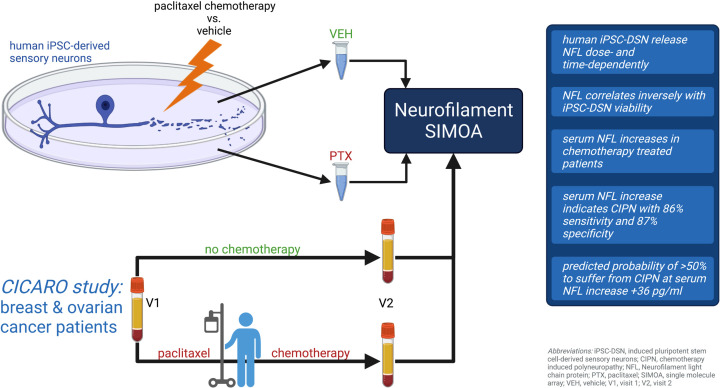

## Introduction

Chemotherapy-induced polyneuropathy (CIPN) is a frequent neurological side effect of cytotoxic chemotherapy. Paclitaxel is used to treat a broad range of solid tumors, including breast and ovarian cancer, and causes CIPN in 57%–82% of patients (reviewed in ref. 1). CIPN strongly affects patients’ quality of life, significantly adds to multimorbidity, and is often dose limiting. Although the symptoms of CIPN typically decline after completion of chemotherapy (2), the majority of paclitaxel-treated cancer survivors still report symptoms of CIPN even years after diagnosis and initial treatment, which are associated with a reduced Karnofsky index and impaired patient-reported physical and psychological well-being (3).

Patients with CIPN and particularly paclitaxel-induced neuropathy develop predominantly sensory symptoms. These entail negative or “minus” symptoms, such as hypoesthesia and hypoalgesia (numbness), but for many patients with CIPN, equally frequent and distressing are positive or “plus” symptoms, such as paresthesia (tingling), temperature sensitivity, allodynia, and pain (4). Negative symptoms are commonly linked to the loss of larger myelinated fibers, whereas positive and pain symptoms are often associated with damage to the smaller (unmyelinated) fibers (5). This means that although clinicians can easily use quantifiable tools to monitor patients for negative symptoms of CIPN, such as vibration sensitivity, the objective assessment of positive symptoms remains challenging in clinical practice: tools such as quantitative sensory testing (QST) are very time-consuming and often not feasible for routine care. In addition, damage to small fibers cannot be detected by nerve conduction studies, and the diagnostic gold standard of small fiber neuropathy requires an invasive skin biopsy. Therefore, the clinical need for an easily accessible and objective biomarker for CIPN is evident.

Neurofilament (NF) proteins make up an essential part of the cytoskeleton in peripheral and central nervous system neurons and function as the structural backbone of axons (6). As axons degrade, an increase of NF, particularly the NF light chain (NFL), is observed in the cerebrospinal fluid (CSF) of patients with diverse neurodegenerative diseases (7). Recently, the development of the ultrasensitive single-molecule array technology has enabled the detection of very low NF concentrations, for instance, as present in serum. Serum NFL (NFL_s_) concentrations were previously shown to correlate with axonal damage and to predict activity in several diseases of the CNS (8, 9), but the value of using NFL_s_ to monitor damage of the peripheral nervous system is less established (10).

To this date, our understanding of the pathomechanisms underlying CIPN is still incomplete. The generation of human induced pluripotent stem cells (hiPSCs) (11) and their differentiation into hiPSC-derived sensory neurons (hiPSC-DSNs) offers the opportunity to study CIPN mechanisms in otherwise inaccessible human cells. Recently, we and others have shown that paclitaxel exposure is associated with morphological signs of axonal damage in hiPSC-DSNs, a dose- and time-dependent decrease of hiPSC-DSN viability, and an upregulation of neuronal injury markers as well as differentially expressed metabolic pathways (12, 13). Because NFL is an axonal protein and CIPN leads to a primarily axonal polyneuropathy, we hypothesized that injured peripheral neurons release NFL, which subsequently leads to increased NFL concentrations. We first investigated this hypothesis in cultured hiPSC-DSNs exposed to paclitaxel and subsequently in patients undergoing paclitaxel chemotherapy, showing that NFL holds the potential to function as a translational biomarker for the detection of neuroaxonal damage in vitro and the diagnosis of CIPN in patients.

## Results

### Assessment of paclitaxel-induced neuroaxonal damage in cultured human sensory neurons.

hiPSC-DSNs from 3 donors were differentiated according to an established protocol with an 11-day phase of differentiation and at least 30 days of maturation (12, 14, 15) (Supplemental Figure 1A; supplemental material available online with this article; https://doi.org/10.1172/jci.insight.154395DS1). Differentiation was commenced when 70%–90% confluence of hiPSCs was reached (Supplemental Figure 1B). In the early maturation phase, bipolar-like neurons were observed (Supplemental Figure 1C), which after further maturation formed morphologically connected ganglia-like structures (Supplemental Figure 1D). Human iPSC-DSN subsets expressed typical neuronal markers, such as beta-III tubulin (Supplemental Figure 1E); markers of the peripheral nervous system, such as peripherin (Supplemental Figure 1F); and markers of the sensory nervous system, such as transient receptor potential cation channel subfamily A member 1 (TRPA1, Supplemental Figure 1G), subfamily V member 4 (TRPV4, Supplemental Figure 1H), or subfamily M member 8 (TRPM8, Supplemental Figure 1I). Calcium imaging experiments revealed functional nociceptor responses upon stimulation with icilin, capsaicin, and ATP, which are agonists of TRPM8, the transient receptor potential cation channel subfamily V member 1 (TRPV1), and P2X/P2Y purinoreceptors. TRPM8 and TRPV1 play a role in the sensation of cold or heat, and purinoreceptors have been implicated in inflammatory pain (Supplemental Figure 1J). The expression of nociceptor markers, such as SCN9A, TRPV1, TRPM8, P2RX3, and Piezo2, was confirmed using RNA-Seq in all 3 hiPSC-DSN cell lines as described previously (12, 16). Purity of the hiPSC-DSN cell lines was investigated using FACS, confirming that early d15 hiPSC-DSNs already expressed on average 99.4% ± 0.9% beta-III tubulin and 88.7% ± 6.4% peripherin; 85.6% ± 7.8% of hiPSC-DSNs expressed both markers (Supplemental Figure 1, K and L, and Supplemental Table 1).

**Figure 1:**
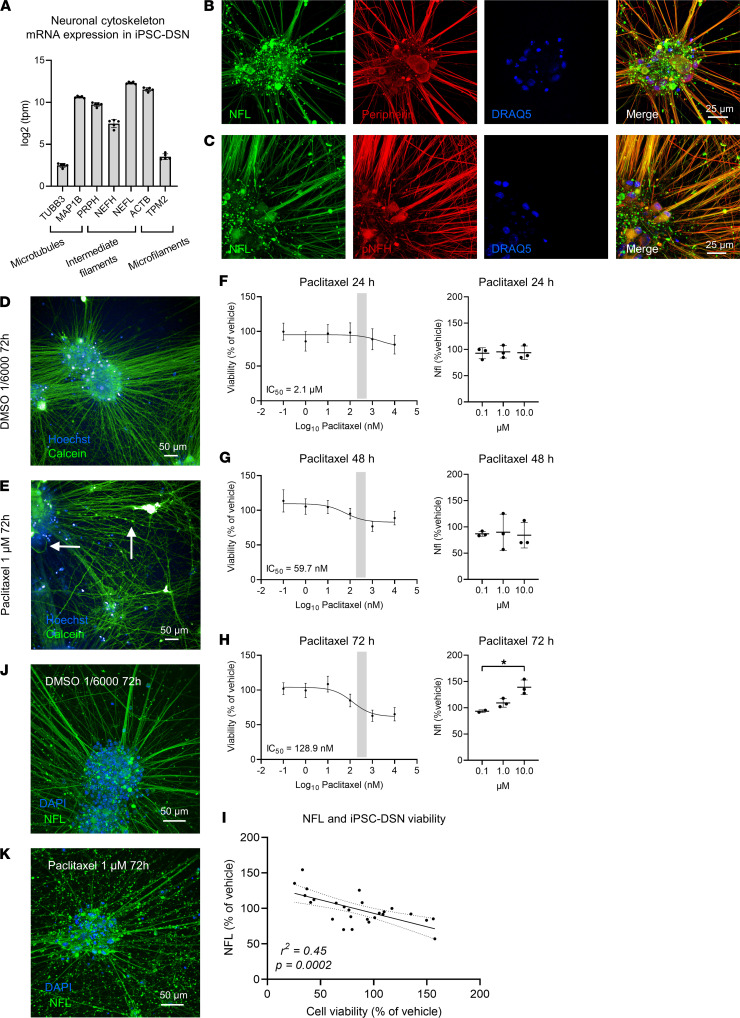
Neurofilament proteins and viability in human iPSC-DSNs treated with paclitaxel. (**A**) Human induced pluripotent stem cell–derived sensory neurons (hiPSC-DSNs) express cytoskeleton protein mRNA. (**B** and **C**) Immunocytochemistry of the cytoskeleton proteins peripherin, NFL, and phosphorylated NF heavy chain (pNFH) indicate colocalization of peripherin with NFL and NFL with pNFH (scale bar: 25 μm). (**D** and **E**) In comparison to vehicle (DMSO), treatment with paclitaxel at 1 μM for 72 hours led to axonal blebbing (**E**, vertical arrow) in living cells and apoptosis (**E**, horizontal arrow) (scale bar: 50 μm) (see also Supplemental Figure 2). (**F**–**H**) A time- and dose-dependent decrease in hiPSC-DSN viability (mean with 95% CI) and a corresponding increase of NFL in the supernatant (mean ± SD) was observed upon paclitaxel incubation. (**I**) Human iPSC-DSN viability and NFL concentrations in the supernatant correlated inversely in response to 72-hour paclitaxel incubation. (**J** and **K**) Axonal NFL expression diminished and concentrated in cytoskeletal debris in response to paclitaxel treatment compared with vehicle-treated hiPSC-DSN (scale bar: 50 μm). Statistical analysis: (**F**–**H**, left column) nonlinear regression (log-inhibitor vs. response, 3 parameters) of data from *n* = 9 independent experiments; (**F**–**H**, right column) Kruskal-Wallis test of data from *n* = 9 independent experiments; (**I**) Pearson’s correlation from *n* = 9 independent experiments of 24- to 72-hour paclitaxel-treated neurons (for details, refer to Supplemental Methods). *P < 0.05.

In the next step, we investigated whether hiPSC-DSNs express mRNA of the neuronal cytoskeleton. RNA-Seq revealed that mRNA of neuronal cytoskeleton proteins, as present in microtubules, intermediate filaments, and microfilaments, was detected in hiPSC-DSNs (Figure 1A). The presence of the intermediate filaments peripherin, NFL, and phosphorylated NF heavy chain could be verified with immunocytochemistry in hiPSC-DSNs (Figure 1, B and C). Using calcein live-cell imaging, we observed that incubation of hiPSC-DSNs with 1 μM paclitaxel for 72 hours led to axonal blebbing as an early sign of axonal injury as well as apoptotic cells, while hiPSC-DSNs treated with vehicle (DMSO) remained intact (Figure 1, D and E, and Supplemental Figure 2, A–E). We then investigated hiPSC-DSN viability and cytotoxicity upon paclitaxel exposure at different concentrations and for various durations. Exposure of hiPSC-DSNs to paclitaxel for 24 hours led to a modest decrease of viability only at higher concentrations of paclitaxel (IC_50_ = 2.1 μM; 100 nM: 98.3% ± 26.3% of vehicle; 1 μM: 88.7% ± 28.4% of vehicle; 10 μM: 80.9% ± 23.4% of vehicle, Figure 1F), and 48 hours of exposure resulted in an IC_50_ close to the steady-state concentration necessary for clinical treatment in patients (IC_50_ = 59.7 nM; 100 nM: 95.2% ± 13.9% of vehicle, 1 μM: 76.8% ± 13.6% of vehicle, Figure 1G). Effects were more pronounced when paclitaxel was applied for 72 hours, leading to a dose-dependent decline of hiPSC-DSN viability in clinically relevant paclitaxel concentrations (IC_50_ = 128.9 nM; 100 nM: 85.0% ± 16.5% of vehicle; 1 μM: 63.0% ± 15.3% of vehicle; 10 μM: 65.2% ± 17.3% of vehicle, Figure 1H). These observations corresponded with a time- and dose-dependent increase of NFL concentrations in the cell culture supernatants, with small effects in hiPSC-DSNs exposed to paclitaxel for 24–48 hours, but robust effects upon 72 hours of exposure (NFL at 1 μM: 109% ± 8% of vehicle, NFL at 10 μM: 139% ± 14% of vehicle, Kruskal-Wallis test, *P* = 0.042, Figure 1, F–H). Next, we evaluated the correlation of hiPSC-DSN viability with NFL concentrations. As expected, NFL concentrations in the supernatants correlated inversely with hiPSC-DSN viability (Figure 1I). We then assessed NFL immunoreactivity morphologically after 72 hours of treatment of hiPSC-DSNs. In response to 1 μM paclitaxel but not vehicle treatment, axonal NFL immunoreactivity diminished while axonal debris stained increasingly positive for NFL fragments outside of the cells (Figure 1, J and K). Additionally, in comparison to vehicle-treated hiPSC-DSNs, paclitaxel incubation at 1 μM for 72 hours was associated with axonal thinning (unpaired 2-tailed *t* test, *P* = 0.031, Supplemental Figure 2C), an increased axonal damage index (unpaired 2-tailed *t* test, *P* = 0.028, Supplemental Figure 2D), and a tendency toward more fragments per total axon area in paclitaxel-treated hiPSC-DSNs (unpaired 2-tailed *t* test, *P* = 0.16, Supplemental Figure 2E), which corroborates the clinical finding of a (sensory) axonopathy. In summary, these in vitro findings of paclitaxel-induced neurotoxicity indicate that paclitaxel leads to time- and dose-dependent neuroaxonal damage with subsequent release and increase of NFL.

### Characteristics of patients with cancer undergoing paclitaxel chemotherapy.

**Figure 2:**
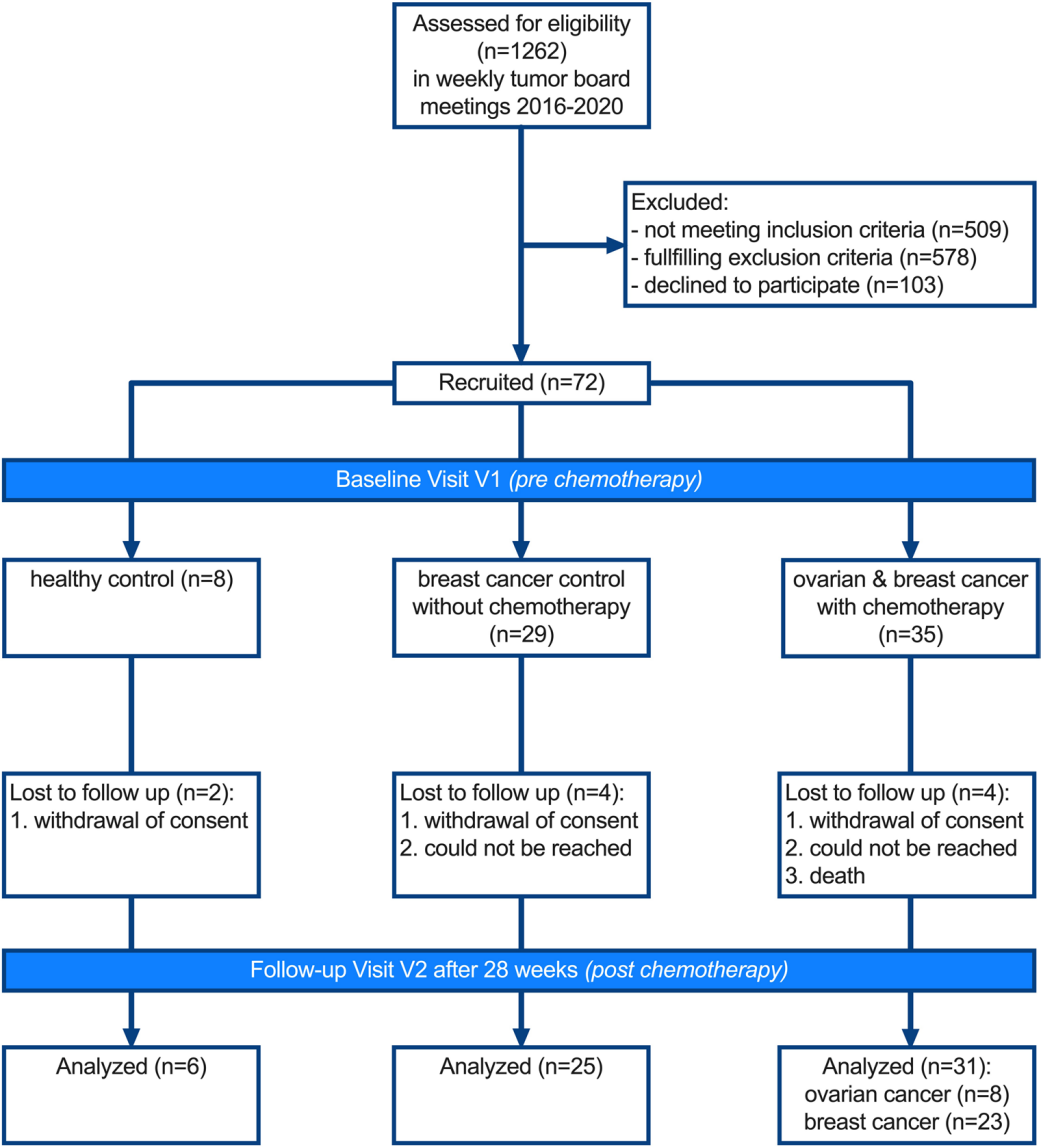
CONSORT diagram of the CICARO trial. Patients were screened in weekly interdisciplinary tumor board meetings and eligibility criteria checked. Eligible patients were contacted by phone regarding possible study participation and, if interested, scheduled for a baseline visit V1, where written informed consent was obtained prior to study inclusion and procedures. Follow-up study visit V2 was scheduled at least 2 weeks after the last chemotherapy application or approximately 6 months after V1 for control patients. A total of *n* = 10 patients were lost to follow-up (i.e., could not be reached, withdrawal of consent, death) and were excluded from the final analysis.

**Figure 3:**
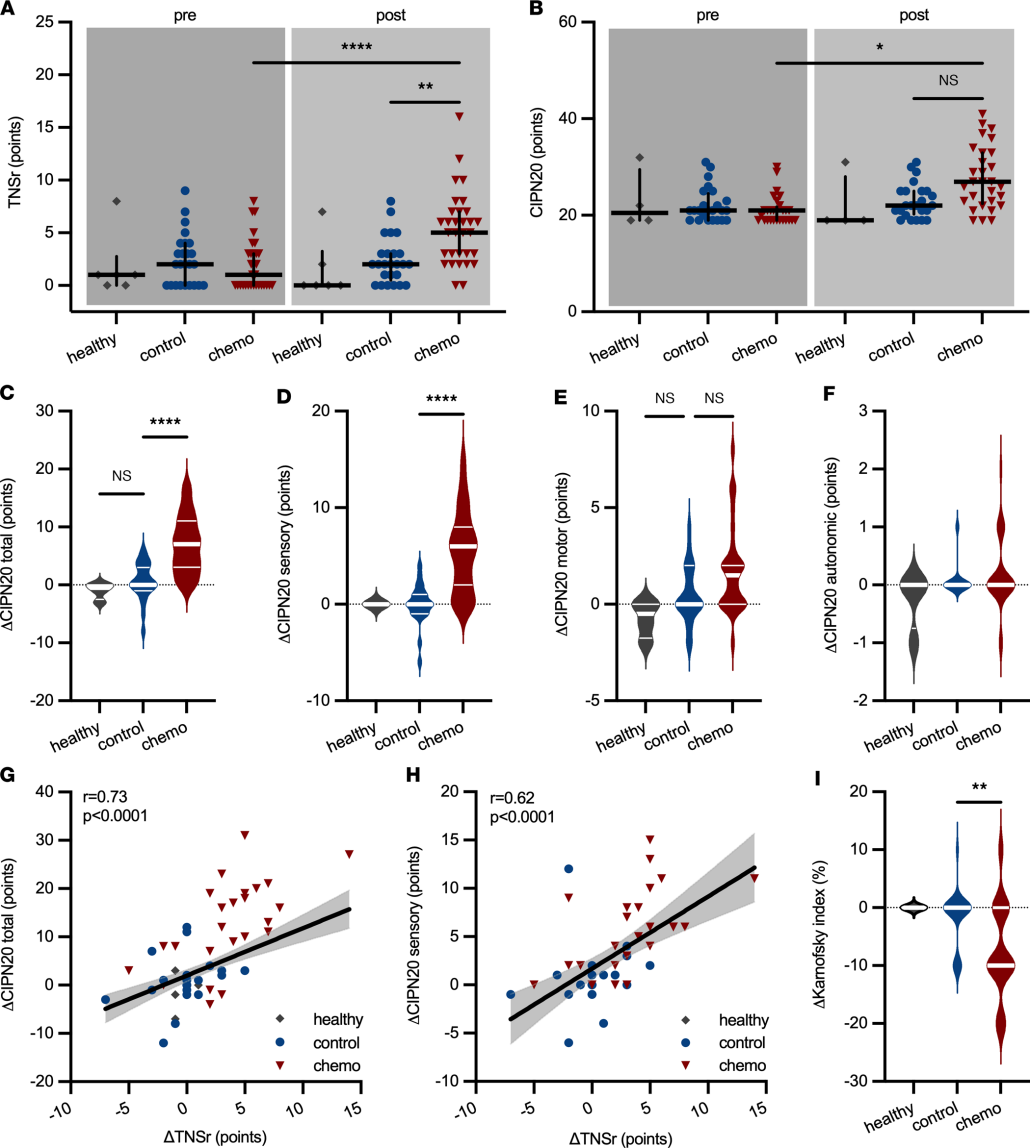
Clinical and patient-reported characteristics of CIPN in patients. (**A**) Chemotherapy but not control patients developed an increase in the Total Neuropathy Score reduced (TNSr). (**B**) Patient-reported symptoms of CIPN, assessed with the EORTC-CIPN20 questionnaire, increased in chemotherapy-treated patients. (**C**) The increase in subjective CIPN symptoms was significantly greater in chemotherapy than control patients, (**D**) particularly for the sensory symptoms of CIPN assessed with the questionnaire, whereas (**E**) only a slight nonsignificant increase was observed for motor symptoms and (**F**) autonomic symptoms. (**G**) Change in subjective CIPN symptoms correlated well with the increase in TNSr, which (**H**) was also the case for only the sensory items of the CIPN20 questionnaire (area filling indicates 95% CI). (**I**) The Karnofsky performance index decreased in chemotherapy-treated patients but not in controls. Statistical analysis: (**A**–**F** and **I**) Kruskal-Wallis test with Dunn’s post hoc correction; (**G** and **H**) linear regression with Spearman’s correlation. Study participants: (**A** and **I**) *n* = 6 (healthy), *n* = 25 (control), *n* = 31 (chemo); (**B**–**H**) *n* = 4 (healthy), *n* = 24 (control), *n* = 29 (chemo). *P < 0.05, **P < 0.01, ****P < 0.0001.

In a next step, we investigated whether an increase of NFL_s_ could also be observed in patients who develop CIPN upon treatment with paclitaxel. CIPN development was tracked in patients enrolled in the longitudinal CICARO cohort study (ClinicalTrials.gov NCT02753036) by a multimodal clinical phenotyping of these patients before and after chemotherapy. A total of *n* = 72 patients were recruited in the CICARO study to 1 of 3 cohorts: otherwise healthy women, who underwent minor gynecological laparoscopic surgery for benign tumors (healthy control group); female patients with breast cancer without chemotherapy (tumor control group); and female patients with ovarian or breast cancer treated with paclitaxel with or without carboplatin (chemo group) and tested from January 2016 (first patient in) to September 2020 (last patient out). *n* = 10 patients were lost to follow-up at visit 2 (V2) and subsequently excluded from the final analysis. Figure 2 summarizes the trial flow. Patients’ clinical and tumor characteristics are displayed in Table 1. CIPN development was assessed with the validated Total Neuropathy Score reduced (TNSr) (17). Patients receiving chemotherapy showed increased TNSr values after treatment compared with tumor control patients without chemotherapy and healthy controls (healthy: 0 ± 3 points [95% CI 0 to 7], control: 2 ± 2 points [95% CI 1 to 3], chemo: 5 ± 4 points [95% CI 3 to 6], Kruskal-Wallis test, *P* = 0.003, Figure 3A). Patient-reported symptoms of CIPN were assessed with the validated European Organization for Research and Treatment of Cancer–CIPN20 questionnaire (18, 19). Patients receiving chemotherapy reported increased symptoms of CIPN after treatment compared with before (pre-control: 21 ± 4 points [95% CI 19 to 23], post-control: 22 ± 4 points [95% CI 21 to 25], pre-chemo: 21 ± 3 points [95% CI 19 to 21], post-chemo: 27 ± 6 points [95% CI 23 to 31], Kruskal-Wallis test, *P* = 0.04, Figure 3B). The increase from baseline was significantly greater in patients receiving chemotherapy compared with control patients (healthy: Δ –0.5 ± 1 points [95% CI –3 to 0], control: Δ 0 ± 3 points [95% CI 0 to 3], chemo: Δ +7 ± 5 points [95% CI 3 to 10], Kruskal-Wallis test, *P* < 0.0001, Figure 3C), which was mostly due to an increase in sensory symptoms (healthy: Δ 0 ± 0 points [95% CI 0 to 0], control: Δ 0 ± 2 points [95% CI –1 to 1], chemo: Δ +6 ± 4 points [95% CI 2 to 7], Kruskal-Wallis test, *P* < 0.0001, Figure 3D) but not motor or autonomic symptoms (Figure 3, E and F). Patient-reported symptoms of CIPN correlated well with the TNSr (linear regression with Spearman’s correlation, *r* = 0.73, *P* < 0.0001, Figure 3G). Again, this was particularly the case for the sensory items of the questionnaire (linear regression with Spearman’s correlation, *r* = 0.63, *P* < 0.0001, Figure 3H). Additionally, the Karnofsky performance index declined more strongly in chemotherapy-treated patients compared with control patients (healthy: Δ 0 ± 0% [95% CI 0 to 0], control: Δ 0 ± 5% [95% CI 0 to 0], chemo: Δ –10 ± 8% [95% CI –10 to 0], Kruskal-Wallis test, *P* = 0.005, Figure 3I).

**Figure 4:**
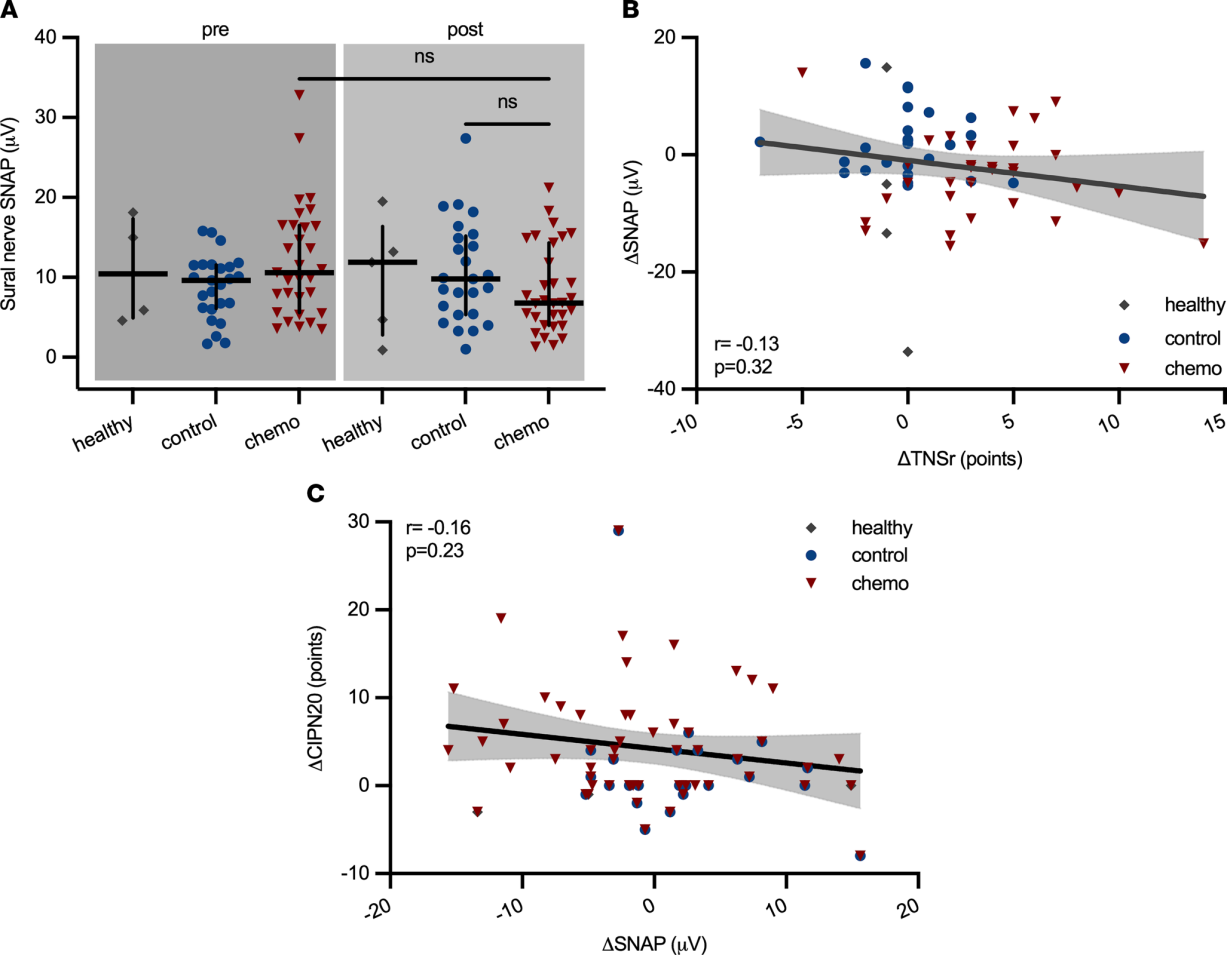
Electrophysiological changes in patients with CIPN. (**A**) The sural nerve sensory nerve action potential (SNAP) amplitudes decreased slightly in patients undergoing chemotherapy, but not controls. (**B** and **C**) Changes in SNAP amplitudes neither correlated with changes in the TNSr nor with patient-subjective symptoms of CIPN (area filling indicates 95% CI). Statistical analysis*:* (**A**) Kruskal-Wallis test; (**B** and **C**) linear regression with Spearman’s correlation. Study participants: (**A** and **B**) *n* = 6 (healthy), *n* = 25 (control), *n* = 31 (chemo); (**C**) *n* = 4 (healthy), *n* = 24 (control), *n* = 29 (chemo).

As part of the TNSr, we also assessed the sensory nerve action potential (SNAP) of the sural nerve and the compound motor action potential of the peroneal nerve along with the respective conduction velocities. The sural nerve SNAP amplitudes tended to decline in the chemotherapy group compared with control patients with cancer who did not receive chemotherapy (healthy: 11.9 ± 7.3 μV [95% CI 0.9 to 19.1], control: 9.8 ± 6.3 μV [95% CI 6.4 to 13.9], chemo: 6.8 ± 5.4 μV [95% CI 5.3 to 9.3], Kruskal-Wallis test, *P* = 0.09, Figure 4A), indicative of an axonal sensory neuropathy. The sensory conduction velocity as well as the peroneal nerve compound motor action potential amplitudes and motor conduction velocity remained unchanged in all groups and over time (data not shown).: However, the change in SNAP amplitude did not correlate with the TNSr (linear regression with Spearman’s correlation, *r* = –0.13, *P* = 0.32, Figure 4B), nor did the SNAP amplitude correlate with patient-reported symptoms of CIPN (Spearman’s *r* = –0.16, *P* = 0.23, Figure 4C). In conclusion, the majority of paclitaxel-treated patients in the CICARO cohort experienced significant symptoms of CIPN as assessed by the validated TNSr. Nerve conduction studies only partially reflected CIPN, most likely because small fiber damage — a common symptom of CIPN — was missed by these examinations. Additionally, the interrater and intrarater variability inherent to nerve conduction studies limits their usage for longitudinal assessment (20).

### NFL_s_ concentrations in chemotherapy-treated patients with and without CIPN.

**Figure 5:**
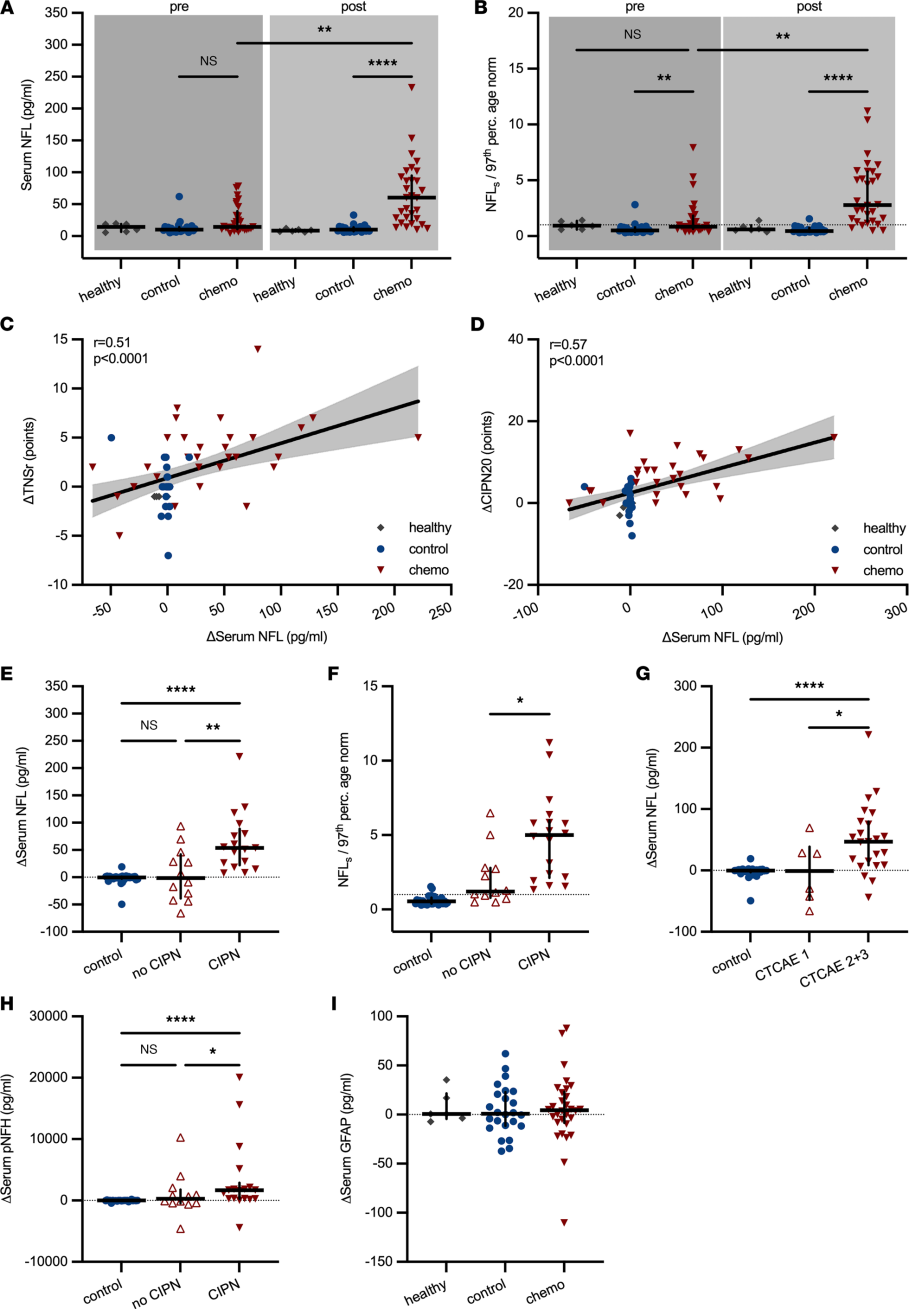
Serum neurofilament concentrations in control and chemotherapy-treated patients. (**A**) NFL_s_ concentrations were significantly higher in patients treated with chemotherapy (chemo) than patients who did not receive chemotherapy (control). (**B**) The age-adjusted upper limit of normal (97th percentile) for NFL_s_ concentrations was calculated as NFL_norm_ = 4.19 × 1.029^age^ (21). Patients’ median NFL_s_ concentrations were below age-adjusted upper normal values at baseline in all groups. NFL_s_ values increased 2.8-fold over the age-adjusted upper limit of normal in chemotherapy-treated patients but not controls after treatment (dotted line marks ratio of 1). (**C**) The change in TNSr and NFL_s_ correlated positively as did (**D**) the increase in patient-reported CIPN symptoms and NFL_s_ (area filling indicates 95% CI). (**E**) The increase in NFL_s_ was particularly observed in chemotherapy-treated patients, who developed clinically significant CIPN (defined as ΔTNSr ≥ 3 points) compared with chemotherapy-treated patients without CIPN and control patients. (**F**) The same was true for age-adjusted NFL_s_ concentrations. (**G**) Increased NFL_s_ values were also observed in chemotherapy-treated patients stratified according to CTCAE grades for peripheral sensory neuropathy: on average grade 2 and 3 CIPN (*n* = 25) resulted in a significant rise in NFL_s_ values compared with asymptomatic grade 1 (*n* = 6) and controls (*n* = 30). (**H**) Higher serum concentrations of phosphorylated NF heavy chain (pNFH) concentrations were observed in patients with CIPN. (**I**) No changes in GFAP as indicator of CNS glial cell (astrocyte) damage were seen in any group. Statistical analysis: (**A**, **B**, and **E**–**I**) Kruskal-Wallis test; (**C** and **D**) linear regression with Spearman’s correlation. Study participants: (**A**–**C** and **I**) *n* = 6 (healthy), *n* = 24 (control), *n* = 30 (chemo); (**D**) *n* = 4 (healthy), *n* = 24 (control), *n* = 29 (chemo); (**E**, **F**, and **H**) *n* = 30 (control), *n* = 12 (no CIPN), *n* = 17 (CIPN). *P < 0.05, **P < 0.01, ****P < 0.0001.

NFL_s_ concentrations were measured at baseline (visit 1, V1) and after 28 (range 14 to 45) weeks (V2, after chemotherapy) in all participants with ultrasensitive single-molecule array technology. NFL_s_ concentrations at baseline were comparable across all groups and, with the exception of 6/8 patients with ovarian cancer, well below the age-adjusted upper limit of normal (97th percentile; ref. 21). At V2 after chemotherapy, we observed significantly higher NFL_s_ concentrations in chemotherapy-treated patients compared with the control group (healthy: 8.5 ± 2.1 pg/mL [95% CI 6.2 to 12.2], control: 10.3 ± 5.8 pg/mL [95% CI 7.8 to 12.6], chemo: 60.3 ± 50.4 pg/mL [95% CI 29.2 to 86.3], Kruskal-Wallis test, *P* < 0.0001, Figure 5A). In the chemotherapy group, 84% of measured NFL_s_ values were above the individual age-adjusted upper limit of normal after chemotherapy (calculated as ratio of patient NFL_s_/97th percentile age-adjusted norm), whereas in control patients only 6% were (healthy [V2]: 0.6 ± 0.4 fold-change [95% CI 0.4 to 1.4], control [V2]: 0.5 ± 0.3 fold-change [95% CI 0.4 to 0.6], chemo [V2]: 2.8 ± 2.8 fold-change [95% CI 1.6 to 5.1], Kruskal-Wallis test, *P* < 0.0001, Figure 5B). Change in TNSr correlated positively with the increase in NFL_s_ (linear regression with Spearman’s correlation, *r* = 0.51, *P* < 0.0001, Figure 5C) as did patient-reported symptoms of CIPN with ΔNFL_s_ (linear regression with Spearman’s correlation, *r* = 0.57, *P* < 0.0001, Figure 5D).

Because we observed a median increase of +3 TNSr points from baseline in chemotherapy-treated patients, we conservatively categorized patients with ΔTNSr of 3 points or more (19/31 [61%]) as patients with clinically relevant CIPN and chemotherapy-treated patients with ΔTNSr greater than 3 points as patients without CIPN (12/31 [39%]). This conservative approach likely underestimated the number of “true” patients with CIPN because positive symptoms from small fiber damage only account for 1/7 categories in the TNSr. Still, patients with CIPN showed a significantly stronger increase in NFL_s_ compared with patients in the control group and patients without CIPN (control: Δ –0.2 ± 10.2 pg/mL [95% CI –1.3 to 0.8], no CIPN: Δ –1.4 ± 49 pg/mL [95% CI –42.2 to 46.2], CIPN: Δ +53.9 ± 54.4 pg/mL [95% CI 26.2 to 79.6], Kruskal-Wallis test, *P* = 0.001, Figure 5E), which was also the case for the individual ratio of patient NFL_s_ to age-adjusted upper limit of normal as a measure of pathological NFL_s_ values (control: 0.5 ± 0.3 fold-change [95% CI 0.4 to 0.6], no CIPN: 1.2 ± 1.9 fold-change [95% CI 0.7 to 2.8], CIPN: 5.0 ± 2.9 fold-change [95% CI 2.2 to 5.8], Kruskal-Wallis test, *P* = 0.04, Figure 5F). In addition, we observed the same results for ΔNFL_s_ when chemotherapy-treated patients were stratified according to the less-sensitive Common Terminology Criteria for Adverse Events (CTCAE) for peripheral sensory neuropathy (control: Δ –0.2 ± 10.2 pg/mL [95% CI –1.3 to 0.8], asymptomatic CTCAE grade 1 [6/31, 19%]: Δ –0.9 ± 51.8 pg/mL [95% CI –66.0 to 69.7], CTCAE grade 2 [24/31, 78%], and CTCAE grade 3 [1/31, 3%]: Δ +46.7 ± 57 pg/mL [95% CI 14.9 to 75.4], Kruskal-Wallis test, *P* = 0.049, Figure 5G). Interestingly, phosphorylated NF heavy chain also increased in patients with CIPN compared with controls and patients without CIPN, but variations were much higher (control: Δ –7.4 ± 107.4 pg/mL [95% CI –31.3 to 5.2], no CIPN: Δ +265 ± 3525 pg/mL [95% CI –428 to 2063], CIPN: Δ +1654 ± 5939 pg/mL [95% CI 288 to 2149], Kruskal-Wallis test, *P* = 0.04, Figure 5H). On the contrary, glial fibrillary acidic protein (GFAP) — a marker for CNS glial cell (astrocyte) damage — remained largely unchanged across all groups (healthy: Δ +0.5 ± 16.1 pg/mL [95% CI –7.3 to 35.2], control: Δ +0.9 ± 25.2 pg/mL [95% CI –11 to 20.7], chemo: Δ +4.4 ± 36 pg/mL [95% CI –3.6 to 13.8], Kruskal-Wallis test, *P* > 0.99, Figure 5I).

### NFL_s_ as a diagnostic marker of CIPN.

**Figure 6:**
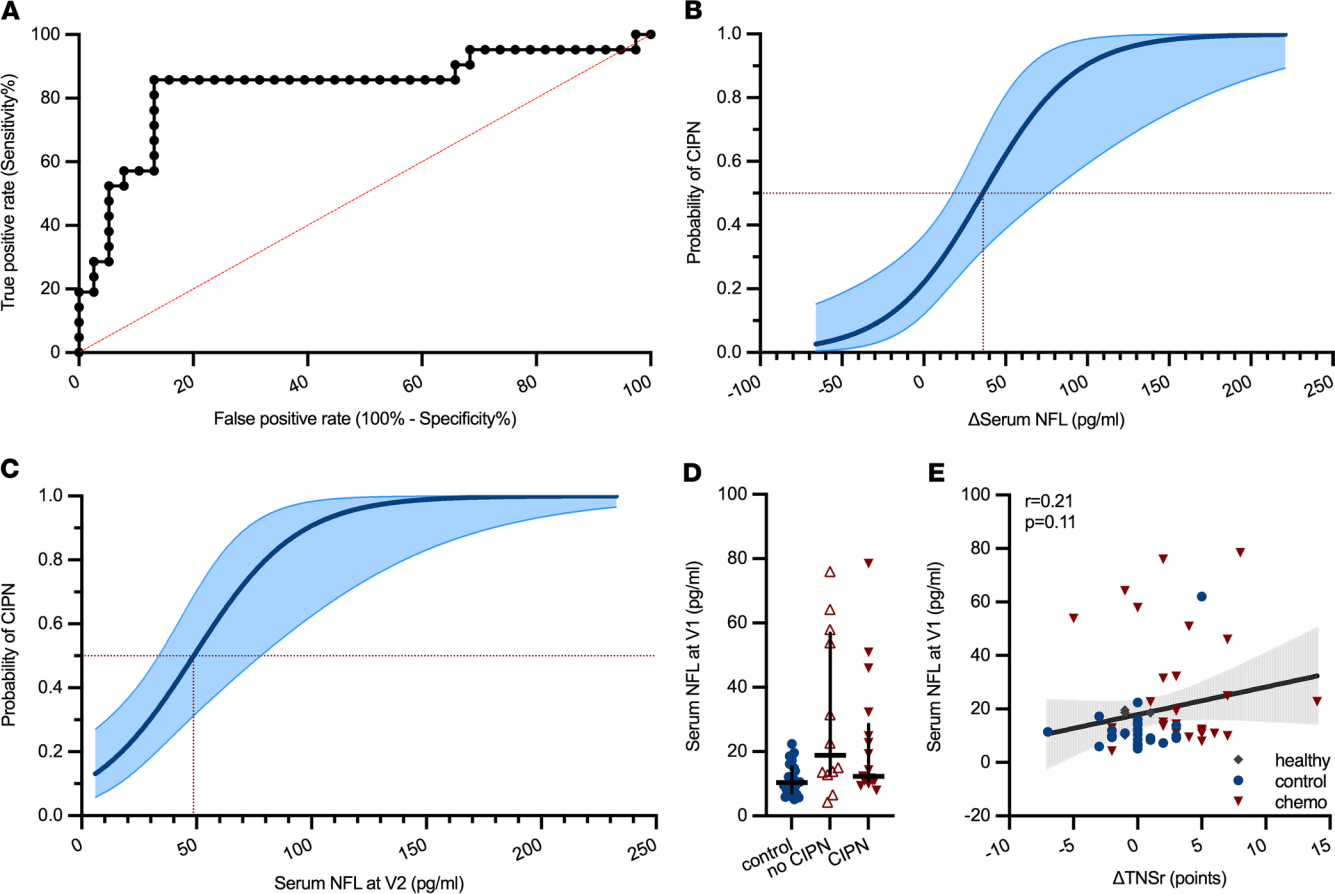
Predicted probability of CIPN diagnosis dependent on NFL_s_ concentrations. (**A**) ROC analysis revealed an 86% sensitivity and 87% specificity for the parameter ΔNFL_s_ greater than 7.05 pg/mL to detect CIPN. (**B**) A likelihood of more than 0.5 for a patient to have CIPN was given at an increase in NFL_s_ by +36 pg/mL (area filling indicates 95% CI). (**C**) Not taking baseline NFL_s_ values into account, a probability of more than 0.5 for a CIPN diagnosis was predicted at NFL_s_ concentrations of 49 pg/mL at V2 (area filling indicates 95% CI). (**D**) Baseline NFL_s_ concentrations were not different among controls, chemotherapy-treated patients without CIPN, and patients with CIPN. (**E**) Baseline NFL_s_ values did not correlate with change in TNSr (area filling indicates 95% CI). Statistical analysis: (**A**) ROC analysis; (**B** and **C**) logistic regression; (**D** and **E**) Kruskal-Wallis test. Study participants: (**A**–**C** and **E**) *n* = 6 (healthy), *n* = 24 (control), *n* = 30 (chemo); (**D**) *n* = 30 (control), *n* = 12 (no CIPN), *n* = 17 (CIPN).

Next, we were interested in the diagnostic properties of NFL_s_. A receiver operating characteristic (ROC) analysis revealed an 86% sensitivity and 87% specificity for an increase of NFL_s_ of +7.05 pg/mL (ΔNFL_s_) in the diagnosis of CIPN (Figure 6A). However, for clinicians, the data from ROC analysis can be difficult to extrapolate to an individual patient’s situation. Therefore, we performed logistic regression analysis to generate a curve of the predicted probability for a patient in the CICARO cohort to have CIPN: an increase in NFL_s_ of +36 pg/mL from baseline (ΔNFL_s_) was associated with a predicted probability of more than 50% for an individual to have CIPN (Figure 6B). Disregarding baseline values and only using NFL_s_ concentrations at V2 after chemotherapy, the likelihood of more than 50% that a patient had CIPN was given at an absolute NFL_s_ concentration of 49 pg/mL (logistic regression, Figure 6C). We also investigated whether baseline NFL_s_ concentrations were associated with future CIPN development. Baseline NFL_s_ values did not correlate with CIPN development: baseline NFL_s_ concentrations did not differ among controls and patients who did or did not later develop CIPN (Figure 6D), and baseline NFL_s_ values did not correlate with future change in TNSr (Figure 6E). We also did not find any signs of increased toxicity in patients with the combination therapy of paclitaxel/carboplatin as ΔTNSr values were similar (Supplemental Figure 3A). Lower changes in NFL_s_ levels in the paclitaxel/carboplatin group (Supplemental Figure 3B) were potentially masked by higher NFL_s_ baseline levels in patients with ovarian cancer, who were exclusively treated with paclitaxel/carboplatin combination therapy, than patients with breast cancer, which we attributed to effects from the extensive surgery (Supplemental Figure 3, C and D).

Because results of our diagnostic tests for NFL_s_ might potentially be skewed because of the higher NFL_s_ baseline values in patients with ovarian cancer, we performed additional ROC and logistic regression analysis in the subcohort of patients who only received paclitaxel monotherapy, which effectively excluded the patients with ovarian cancer and patients with breast cancer with the combination therapy of paclitaxel/carboplatin. Patients treated with paclitaxel only showed a similar increase in TNSr and CIPN20 score points, indicating CIPN development (Supplemental Figure 4, A and B). NFL_s_ and ΔNFL_s_ levels were slightly higher in patients treated with paclitaxel only, as previously observed in the analysis of the entire cohort (Supplemental Figure 4, C and D). However, in the diagnostic tests (ROC and logistic regression analysis), very similar results were observed: the sensitivity increased to 100% while specificity remained at 85% for an equal cutoff value of +7 pg/mL ΔNFL_s_. The threshold of ΔNFL_s_ for a predicted probability of more than 50% to have CIPN was almost identical (Supplemental Figure 4, E and F). We repeated the analysis in the breast cancer cohort, which excluded patients with ovarian cancer, but still contained 26% of patients with a combination therapy of paclitaxel/carboplatin. Again, TNSr, CIPN20, and NFL_s_ results were very similar (Supplemental Figure 5, A–C). In the diagnostic tests, the threshold for both ΔNFL_s_ and NFL_s_ at V2, which indicates a predicted probability of more than 50% to have CIPN, was approximately 5 pg/mL higher in this analysis compared with the evaluation of the entire cohort (Supplemental Figure 5, D and E); the sensitivity for ΔNFL_s_ increased to 100% and specificity decreased slightly to 81% (Supplemental Figure 5F). In conclusion, additional analysis of certain subpopulations revealed very similar sensitivity and specificity results and cutoff values for NFL_s_ in the diagnosis of CIPN, which underlines the quality of the presented data.

### CIPN remission after completion of chemotherapy.

Because the range of our V2 examination after chemotherapy was quite large (14 to 45 weeks), we were interested to see how clinical and electrophysiological characteristics change after completion of chemotherapy. Therefore, patients were binned according to their V2 time points to one of the following categories: 14–24 weeks after V1 (median 21 weeks, *n* = 4 [chemo]), 25–34 weeks after V1 (median 28 weeks, *n* = 23 [control] and *n* = 24 [chemo]), and 35 weeks or more after V1 (median 40 weeks, *n* = 3 [control] and *n* = 3 [chemo]). Not surprisingly, the change in TNSr values was highest immediately after chemotherapy completion at 21 weeks and then steadily declined (Supplemental Figure 6A). Similar results were observed for the patient-reported outcome of CIPN (CIPN20 questionnaire, Supplemental Figure 6B). Although not much change in the structural integrity of the sural nerve from baseline was observed at the earliest time point after chemotherapy, the sural nerve SNAP amplitudes declined at 28 and 40 weeks after V1 because the disease takes time to become structurally apparent (Supplemental Figure 6C). Similar results as for the TNSr were observed in the analysis of NFL_s_ levels, which were increased compared with the control at 21 and 28 weeks, but had reached almost normal values at 40 weeks after V1 (Supplemental Figure 6, D and E). Overall, these results — with the exclusion of NFL_s_ measurements, which had not previously been reported in this manner — are in line with data from larger clinical cohorts regarding CIPN remission after chemotherapy completion.

## Discussion

Our data demonstrated that NFL correlated with paclitaxel-induced neuroaxonal damage in vitro. Furthermore, we were able to show that development of CIPN was associated with increases in NFL_s_ and that NFL_s_ correlated with CIPN severity in paclitaxel-treated patients. Our data agree with previous preclinical reports of increased NFL_s_ levels in paclitaxel- and cisplatin-treated rats (22, 23), underlining NFL’s potential as a translational biomarker. NFL_s_ measurements in our study are similar to previously reported data from oxaliplatin-treated patients (24) and recent findings published in a small cohort of patients with breast cancer (25). The latter study could show that NFL_s_ already increased during chemotherapy, but that NFL_s_ did not correlate with findings of chemotherapy-induced cognitive impairment and was heavily influenced by CIPN (25). Since clinical presentation of CIPN may vary and sensory symptoms fluctuate, the challenge of an easily accessible objective parameter to allow early diagnosis of CIPN remains. In contrast to the aforementioned studies in oxaliplatin- and paclitaxel-treated patients (24, 25), our findings demonstrated that based on NFL_s_, a distinction can be made between patients who develop or do not develop clinically significant CIPN, as well as control patients. Even with a conservative cutoff of ΔTNSr of 3 points or more to diagnose CIPN to avoid positive selection bias, NFL_s_ concentrations were much higher in patients with CIPN compared with patients without CIPN. Some of the higher NFL_s_ values in the “no CIPN” group are likely explained by patients with ΔTNSr of 1–2 points, who may still have significant small fiber damage with predominantly positive sensory symptoms, which are underrepresented in the TNSr as they only account for 1/7 items. This is highlighted by the fact that 6/12 patients in our “no CIPN” group among chemotherapy-treated patients still had sensory peripheral neuropathy grade 2 according to CTCAE. Consequently, this also means that the calculated values of NFL_s_ for a predicted CIPN probability greater than 50% were likely overestimated and may in fact be lower as indicated by the ROC analysis. NFL_s_ concentrations before paclitaxel treatment did not predict CIPN development, which is to be expected because the damage has not yet occurred. This is in contrast to other neurological diseases, such as multiple sclerosis (26), vascular dementia (27), or Alzheimer disease (28), in which higher NFL concentrations in asymptomatic yet diagnosed patients are predictive of future disease activity or progression. More importantly than NFL_s_ baseline values, our study modeled predicted probabilities of patients to have CIPN based on (change in) NFL_s_, which aids CIPN diagnosis and supports the argument to implement serial NFL_s_ measurements during chemotherapy in the future to detect and verify CIPN early and expedite neurological care.

Potential limitations of our study are the relatively low number of patients and the confinement to gynecological tumors and therefore women. Although some sex-related differences were found in pain perception in CIPN in preclinical studies in mice, mainly regarding cold and mechanical hypersensitivity (29, 30), severity of CIPN in electrophysiological studies was similar across strains and sex (30). Some clinical studies also indicate that the prevalence and severity of CIPN are not majorly different between the sexes (31). Furthermore, it has to be kept in mind that NFL_s_ is abundant in the peripheral and central nervous system, which raises the possibility that the development of postchemotherapy cognitive impairment (PCCI) may confound the usefulness of NFL_s_ in the context of CIPN. Given that previous studies could not establish correlation of NFL_s_ values with the development of PCCI (25) and we could not observe an increase of GFAP, a marker of astrocyte damage, NFL_s_ released because of CNS neurotoxicity appears to play a minor role, if any at all. Strengths of the study, which in part counteract the low sample size, include the longitudinal design, careful assessment of multifactorial endpoints, inclusion of a non-chemotherapy-treated cancer control group, and the homogeneity regarding the chemotherapy protocols. Future clinical studies with longer follow-up periods should elucidate whether NFL_s_ may also predict patients with CIPN with irreversible long-term damage of the peripheral nervous system as opposed to patients with reversible CIPN symptoms and investigate whether an increase of NFL_s_ occurs before clinical manifestation of CIPN symptoms.

In summary, this study showed that sensory neurons released NFL upon neuroaxonal damage induced by paclitaxel in vitro and strongly suggests the use of (serial) NFL_s_ measurements to assess CIPN in patients. This finding holds the potential to facilitate treatment guidance of patients undergoing chemotherapy, as well as to accelerate development of preventive therapies for CIPN.

## Methods

For a detailed description of the methods, please refer to the Supplemental Methods.

### In vitro experiments.

hiPSC-DSNs were differentiated from the established stem cell line BIHi005-A (https://hpscreg.eu/cell-line/BIHi005-A, Berlin Institute of Health Stem Cell Core Facility), obtained by reprogramming of human dermal fibroblasts using Sendai viral vectors as previously reported (32, 33). After approval by the Charité ethics committee (ClinicalTrials.gov NCT02753036) and written informed consent had been obtained, 2 additional hiPSC lines from 2 patients with breast cancer were reprogrammed from PBMCs using Sendai viral vectors (cell lines BIHi264-A and BIHi263-A, Berlin Institute of Health Stem Cell Core Facility), using established protocols (34). iPSCs were tested for the absence of the reprogramming vector, with immunofluorescence staining for pluripotency markers, in vitro directed differentiation into the 3 germ layers, karyotyping using SNP arrays, and g banding, as described previously (34) and in detail in another study (32). Stem cells were maintained on growth factor reduced Geltrex (Gibco) in E8 media with daily media exchange. Cells were enzymatically clump-passaged when more than 70% confluence was achieved, usually every 2–4 days using UltraPure 0.5 M EDTA (Thermo Fisher Scientific). hiPSC-DSN differentiation was performed as previously described (15), following a protocol of 11 days of differentiation by small molecule inhibition and at least 30 days of further maturation in growth factor–enriched media (14) (Supplemental Figure 1A). Differentiation success was confirmed by staining for the expression of peripheral nervous system markers, sensory neuron markers, FACS analyses for hiPSC-DSN purity, and calcium imaging for functionality (Supplemental Methods). Neurotoxicity in hiPSC-DSNs was assessed as a compound measure of viability and loss of membrane integrity (i.e., imminent death) as previously described (35) and in detail in the Supplemental Methods.

RNA-Seq data displayed in this publication are accessible in National Center for Biotechnology Information’s Gene Expression Omnibus (GSE173610) and in the article by Schinke et al. (16).

### Recruitment and inclusion in clinical trial.

Possible participants were screened in weekly interdisciplinary tumor board meetings, and eligible patients were scheduled for baseline visit V1 before the start of chemotherapy, where written informed consent was obtained from participants prior to inclusion in the study. All participants had to fulfill the following inclusion criteria: a) 18 to 70 years of age, b) Karnofsky index 70% or greater, and c) 8 years of school education or more. Patients with prior neurotoxic chemotherapy, former or current alcohol or drug abuse, mild cognitive impairment or dementia, postsurgery delirium, major depression, or anemia less than 8 g/dL were excluded from participation.

A total of *n* = 72 patients were recruited to 1 of 3 cohorts: otherwise healthy women, who underwent minor gynecological laparoscopic surgery for benign tumors (healthy control group); female patients with breast cancer receiving antihormonal and/or localized radiation treatment but without chemotherapy (tumor control group); and female patients with ovarian and breast cancer treated with paclitaxel with or without carboplatin (chemo group). Included participants were tested from January 2016 (first patient in) to September 2020 (last patient out). A total of *n* = 10 patients were lost to follow-up at V2 (could not be reached, withdrawal of consent, death) and subsequently excluded from the final analysis. Figure 2 summarizes the trial flow.

### Neurological and electrophysiological examination.

A neurological examination was conducted at study visits V1 (baseline, before chemotherapy) and 28 (range 14 to 45) weeks later (V2, after chemotherapy). Vibration sensitivity was measured with a commercial Riedel-Seyfert tuning fork and values of 6/8 or lower were regarded as reduced. Muscle strength was documented according to the Medical Research Council (MRC) scale. Reflex status was rated as normal, reduced, or absent. Motor conduction velocity was measured in one leg by a supramaximal stimulus of the common and deep peroneal nerve proximal of the ankle joint (S1) and under the head of the fibula (S2) by recording the corresponding compound motor action potentials with surface electrodes positioned over the M. extensor digitorum brevis using an Evidence ENG/EMG device (Schreiber & Tholen). Supramaximal serial electric stimuli (at least 20) were applied to the lower calf, and the SNAP and sensory conduction velocity of the sural nerve were measured over the skin inferior to the lateral malleolus with surface electrodes. CIPN development was graded according to the validated TNSr (17).

### NF measurements.

NFL, phosphorylated NF heavy chain, and GFAP were measured by single-molecule array technology (Quanterix) in supernatants collected from hiPSC-DSN cultures (Labor Berlin GmbH) and in patient sera (NMI) by operators following a protocol with blinded clinical data and treatment.

### Statistics.

Patients lost to follow-up were excluded from the analysis. Final analysis was done with data from *n* = 6 healthy controls, *n* = 25 tumor controls, and *n* = 31 patients receiving chemotherapy. Missing data values were not imputed. GraphPad Prism v9 and Stata v16 (StataCorp LLC) were used for statistical analysis and data visualization. Gaussian distribution was checked with a Shapiro-Wilk normality test before statistical analysis. Normally distributed data were analyzed using unpaired 2-tailed *t* tests (2-group comparisons), whereas not normally distributed data were analyzed with Mann-Whitney *U* test (2-group comparison) or Kruskal-Wallis test with Dunn’s method for post hoc adjustment for multiple comparisons. In this study, experiments with hiPSC-DSNs were conducted that originated from *n* = 3 iPSC donors (BIHi005-A, BIHi263-A, BIHi264-A). All experiments on hiPSC-DSN viability/cytotoxicity were replicated at least 3 times (i.e., *n* = 3 independent differentiations) with 4 technical replicates for each condition. In vitro NFL experiments were replicated at least 3 times (i.e., *n* = 3 plates independently matured >30 days) with 4 technical replicates pooled for each condition. In vitro data are reported as mean with 95% CI (viability assays) or mean ± SD (mRNA expression, NFL measurement). Patient data are reported in the text as median ± SD, including 95% CI, and displayed as median with IQR. Nonlinear regression analysis was performed to obtain dose-response curves (log-inhibitor vs. response, 3 parameters). Linear regression was performed with Pearson’s correlation (cell culture data, normal distribution) and with Spearman’s correlation (clinical data, no Gaussian distribution) between 2 variables. Binary multiple logistic regression analysis was used to calculate predicted probabilities of CIPN development. Data are available on Mendeley Data: “Neurofilament proteins as potential biomarker in chemotherapy-induced polyneuropathy,” doi: 10.17632/w7w3myjpgc.1.

### Study approval.

The observational CICARO cohort study was approved by the ethics committee of Charité — Universitätsmedizin Berlin (EA4/069/14, Berlin, Germany) and registered prior to recruitment at ClinicalTrials.gov (NCT02753036). Written informed consent was obtained from all participants prior to inclusion in the study.

## Author contributions

PH, WB, and ME designed the clinical study. PH and WB wrote the clinical trial application and obtained approval for the study. NB, JUB, and JS screened patients. PH, WB, and CS recruited patients. PH, WB, CS, ADD, and JK evaluated patients. CS, SKM, LH, and LN conducted the in vitro experiments. PK analyzed cell culture supernatants. JCG, KR, and CJS analyzed patient sera. PH and CS analyzed the data and compiled figures. PH, WB, and CS wrote the manuscript. All authors reviewed the manuscript. PH, WB, CS, KR, and ME obtained funding for the study. Authorship order for equally contributing first authors (PH and CS) was assigned alphabetically.

## Supplementary Material

Supplemental data

Trial reporting checklists

ICMJE disclosure forms

## Figures and Tables

**Table 1:**
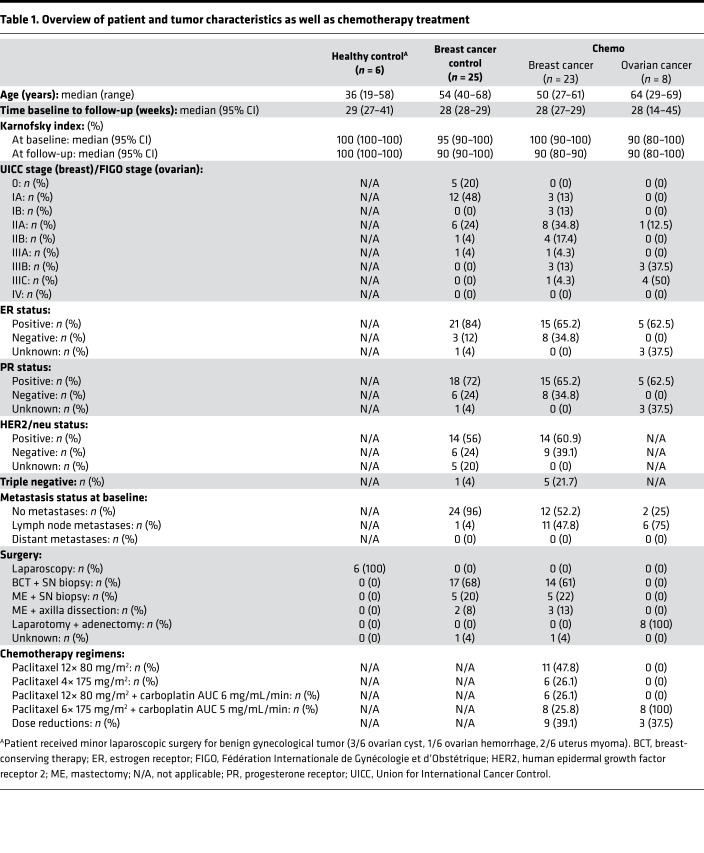
Overview of patient and tumor characteristics as well as chemotherapy treatment

## Version 1 02/08/2022

In-Press Preview

## Version 2 03/22/2022

Electronic publication
