# Supplementary material for: Neurofilament proteins as a potential biomarker in chemotherapy-induced polyneuropathy
Source: JCI Insight. 2022 Mar 22;7(6):e154395. doi: 10.1172/jci.insight.154395 (PMC8986065; doi:10.1172/jci.insight.154395)
Supplement: Supplemental data [file jciinsight-7-154395-s054.pdf]

Supplemental information

Material and methods

In vitro experiments

Induced pluripotent stem cells (iPSC): Human iPSC-derived sensory neurons were differentiated from the established stem cell line BIHi005-A (https://hpscreg.eu/cell-line/BIHi005-A, Berlin Institute of Health Stem Cell Core Facility), obtained by reprogramming of human dermal fibroblasts using Sendai viral vectors as previously reported (1, 2). After approval by the Charité ethics committee (NCT02753036) and written informed consent had been obtained, two additional hiPS cell lines from two breast cancer patients which belonged to the study cohort were reprogrammed from peripheral mononuclear blood cells (PBMC) using Sendai viral vectors (s.c. cell lines BIHi264-A and BIHi263-A, Berlin Institute of Health Stem Cell Core Facility) according to established protocols (3). iPSC were tested for the absence of the reprogramming vector, with immunofluorescence staining for pluripotency markers, in vitro directed differentiation into the three germ layers and karyotyping using SNP arrays and g banding, as described previously (3) and in detail in (4). iPSC were maintained on growth factor reduced Geltrex (Gibco) in E8 media, which was exchanged daily. iPSC were enzymatically clump-passaged every 2-4 days when >70 % confluency was reached using EDTA (UltraPure 0.5 M EDTA, Thermo Fisher).

Human iPSC-derived sensory neurons (iPSC-DSN). Differentiation was performed as previously described (5). In brief, confluent clump-passaged iPSC were single cell seeded at a density between 200.000-400.000/well into 6-well plates (6) coated with growth factor reduced Geltrex (Gibco) and cultured in E8 media until 70-80 % confluency was achieved, which usually took two to three days. *On day 0*, neural induction was initiated by replacing E8 media with Knockout Serum Replacement Media (500 ml DMEM-KO [Gibco]) supplemented with 130ml CTS KnockOut SR

XenoFree Medium (Gibco), 1x MEM-Non-essential Amino Acid Solution (Sigma), 1x Glutamax (Gibco) and 0.01mM β-Mercapto-ethanol (Gibco) containing the two small molecule inhibitors '2i': 100 nM LDN-193189 (Sigma) and 10 µM SB-431542 (Peprotech) for neuroectoderm differentiation. *On day 2*, '3i' consisting of 3 µM CHIR99021 (Sigma), 10 µM DAPT (Sigma) and 10 µM SU5402 (Sigma) were added for neural crest specification. *On day 4*, N2B27 media (500 ml Neurobasal-Medium [Gibco], 5 ml of N-2 (100x) supplement [Gibco], 10 ml of B27-supplement (50x) without Vitamin A [Gibco], 1x Glutamax and 0.01 mM β-Mercapto-ethanol [Sigma]) was progressively phased in. *From day 6 onwards*, '2i' addition was ceased but '3i' continued to be added until Day 11. *On day 11*, iPSC-DSN were reseeded. Cells were dissociated using TrypLe select (Gibco) and resuspended in N2B27 maintenance media containing 10 µM ROCK inhibitor. iPSC-DSN were reseeded at a density of 48.000/well into geltrex-coated 96-well plates (6) for cell viability/cytotoxicity assays and NFL measurement, or geltrex coated coverslips for staining. ROCK inhibitor containing N2B27 media was removed 24h after reseed and replaced with N2B27 supplemented with BDNF, GDNF, βNGF and NT3 (all at 25 ng/ml, Peprotech). *On day 14*, cells were treated with 1 µg/ml Mitomycin C (Sigma) for 2h to eliminate the few dividing non-neuronal cells. iPSC-DSN were maturated for at least 30 more days, and half media change was performed every 3-4 days with growth factor enriched N2B27 media (BDNF, GDNF, βNGF and NT3, all at 25 ng/ml, Peprotech).

Compound preparation: Paclitaxel (Adipogen) was dissolved in DMSO to reach 6 mM stock solutions. Concentrations of the vehicles were 1/600 DMSO (for cell viability experiments) or 1/6000 for automated microscopy. All final solutions were prepared on the day of experimentation.

Neurotoxicity: For cell viability and cytotoxicity, MTT and proteases assays were applied as described previously (7). Paclitaxel was added to iPSC-DSN cultured in 96-

well plates and incubated for 24, 48 and 72h. 50µl of the supernatant were transferred into black 96 well-plates (Thermo Scientific Nunc F96 MicroWell) and quantified by fluorescence measurement applying the Promega Cytotox-Fluor Assay (Promega) and measured in the Promega GloMax Reader (Promega), according to manufacturers' instructions. MTT was added to the wells of the culture plates in a 1:10 ratio. Reaction was stopped after 35min adding sodium dodecyl sulfate (SDS) 10 % in 0.01 M HCl in a 1:2 ratio to the remaining supernatant. Cells were dissolved overnight and absorbance was measured at 560 nm. All values were background subtracted and standardized to percentage of vehicle. For cell viability/cytotoxicity results in Figure 1F-H, 4 technical replicates were pooled, and three biological replicates (3 different plates independently matured >30d, all BIHi-263a) were included in the calculation of each of the Figures in 1F-H, left column (9 experiments in total). For NFL assessment (Figures in 1F-H, right column), 4 technical replicates from the 96-well plates were pooled for each paclitaxel concentration. For each time point from 24-72h, three independent experiments were undertaken (maturation for >30d in different 96-well plates, all derived from iPSC-DSN of the cell line BIHi005-A, resulting in 9 experiments in total which were included in Figure 1F-H, right column). To meet the dynamic range of SIMOA, NFL supernatants were diluted in a 1:250 ratio. In total, 14/384 values from the cell viability/cytotoxicity assays were excluded either because of cell detachment before the experiment or as values exceeded >2 SDs (in total, exclusion of 3.6% of all measures). 1/27 NFL measurements was excluded (Figure 1H + I, 100 pM paclitaxel) because of an NFL concentration which exceeded >2SDs from the rest of the other values (3.7% of data points). The correlation of iPSC-DSN viability with NFL concentrations (Figure 1I) was calculated from the composite measure of live/dead ratio applying the MTT and cytotox fluor assay normalized to vehicle in association to

NFL concentrations normalized to vehicle from the same experiments (all performed in cell line BIHi005-A, 9 experiments in total).

Staining: Neurofilament light chain (NFL) and phosphorylated Neurofilament heavy chain (pNFH) were stained in iPSC-DSN. In brief, cells were fixed with 2 % paraformaldehyde for 15min, rinsed twice and incubated for 1h at room temperature with blocking buffer consisting of 1xPBS, 1 % bovine serum albumin (BSA, Sigma, A2153), 10 % normal goat serum and 0.1 % Triton X. The following primary antibodies were diluted in 1 % BSA: 1:500 Peripherin (rabbit, ThermoFisher, PA1-10018), 1:1000 NFL (Uman Diagnostics, UD2), 1:1000 pNFH (Abcam, ab207176), 1:50 TRPM8 (rabbit, Abcam, ab3243), 1:25 TRPA1 (rabbit, Novus Biologicals, NB110-40763SS), 1:50 TRPV4 (rabbit, LifeSpan, LS-C94498). After incubation for 24h at 4°C, cells were washed 3x with 1x PBS and incubated for 1h at room temperature with 1:600 secondary antibodies diluted in 1 % BSA, using goat anti-rabbit Alexa 488 (Invitrogen, A-11034) or goat anti-mouse Alexa 488 (Invitrogen, A-11029) if single stained, or in combination with goat anti-mouse Alexa 568 (Invitrogen, A-11031) if double-stained, and washed 3x with 1x PBS. Nuclei were stained with 10 µM DRAQ5 (Thermo Scientific, 62251) by 30min incubation, washed once with 1x PBS and mounted with ProLong™ Gold Antifade Mountant DAPI (Molecular Probes, P36931). Stainings were visualized on a Leica TCS SP II with a Leica DFC3000G camera fitted with 10x, 20x, 40x and 63x objectives.

Automated microscopic imaging before and after 72h of exposure to 1 µM paclitaxel: Opera Phenix High Content Screening System (Perkin Elmer, USA) was used with the associated Harmony® Office Software for live cell imaging. Cells were incubated with calcein (in DMSO, Invitrogen, C1430) at 1 µg/ml for 20-30min in N2B27 (prepared as described above, but with Neurobasal Media Phenolred-free, Gibco). Images were taken automatically at pre-defined regions of interest in 1 µm slices with a 20x water

objective, overlaying a maximum projection of 15 images. Predefined regions were manually compared, and representative images selected.

Axonal damage: The axonal damage index was calculated according to Sasaki et al. (8) as the ratio of the area *or* number of axonal fragments in relation to the total surface area covered by axons. 16 live cell images were analyzed (20x, resolution 600x600pixels), 8 from paclitaxel treated iPSC-DSN (1 µM, 72h) and 8 from DMSO treated iPSC-DSN (maturation > d60, all from cell line BIHi 263-A), for each group from 3 different wells and at least three different fields of interest, which fulfilled the prerequisite to primarily depict axons rather than somata. In one image field (1/16 images), an area which was covered by a large diameter iPSC-DSN ganglion was excluded from analyses (manually cut out) to not skew the calculated axon area. Image analyses was undertaken using ImageJ. All images were transformed to an 8-bit format and then binarized using the threshold tool, and the area covered by axons calculated in relation to the full image. To detect axonal degeneration, the particle analyzer tool was applied (settings: 7-60 pixels [set according to previous optimization manually measuring fragment sizes], circularity 0.7-1.0). 1 out of 16 measurements was excluded from analyses as it exceeded >2SDs (vehicle group).

Transcriptome sequencing analyses: Transcriptome analyses were performed as described previously (9). In brief, iPSC-DSN were cultured in geltrex coated 6-well plates at 10^6 cells/well as described above. After maturation until d44 - d49, RNA was harvested with the Aurum™ Total RNA Mini Kit according to manufacturers' instructions. RNA sequencing was performed by Brooks Life Sciences Genewiz® with PolyA selection for RNA removal, 2x150bp sequencing configuration and 20-30 million reads per sample. 5 samples were analyzed. From our publicly available dataset of the transcripts per kilobase million (TPM) published in (10), we searched representative cytoskeleton filaments which were plotted as log2-expression (Figure 1A).

Fluorescence-activated Cell Sorting (FACS) Analysis: D11 iPSN-DSN were thawed and reseeded according to the protocol described in (9, 11) and on d15, they were harvested for flow cytometry using TrypLe select (Gibco), resuspended in N2B27 maintenance media containing 10 µM ROCK inhibitor and strained through a 100 µm cell strainer to obtain single cell suspension. They were washed with FACS wash buffer (0.5 % BSA in PBS and 2 mM EDTA) before fixation and permeabilization using buffers from the FoxP3 Staining Buffer Set (Miltenyi Biotec, 130-093-142), used according to manufacturer's protocol. beta III tubulin (mouse, R&D systems, MAB1195) was diluted 1:125 and peripherin (rabbit, ThermoFisher, PA1-10018) was diluted 1:500 in 1x Permeabilization buffer from the FoxP3 Staining Buffer Set. iPSC-DSN were incubated with intracellular primary antibodies for 30min at room temperature and subsequently washed with 1x Permeabilization buffer before incubating with secondary antibodies. Goat anti-mouse Alexa 488 (Invitrogen, A-11029) and goat anti-rabbit Alexa 635 (Invitrogen, A-31577) were diluted 1:600 in 1x Permeabilization buffer. iPSC-DSN were incubated with secondary antibodies for 30min at room temperature. After incubation, they were washed with 1x Permeabilization buffer and resuspended in 300-400 µl of PBS per tube. Flow cytometry was conducted using a BD FACSCanto II and data was analysed with FlowJo.

Calcium live-cell imaging: Calcium imaging was performed as described previously (9, 12). iPSC-DSN were loaded with Fura-2 AM 5 µM (Life Technologies GmbH, Germany) and 0.02% pluronic F-127 (Life Technologies GmbH, Deutschland) for 30min at 37°C in a standard HEPES buffered solution prepared as 130 mM NaCl, 4.7 mM KCl, 1 mM MgSO4, 1.2 mMKH2PO4, 1.3 mM $CaCl_2$, 20 mM Hepes and 5 mM glucose, pH 7.4. Cell cultures were then washed with standard HEPES buffered solution, and 8-Chamberslides were placed in an Olympus IX 81 microscope equipped with a Uplan objective 20x (Olympus Corporation, Japan).

Fluorescence signals were detected by a cooled CCD-camera at 1 Hz, and data processed using Xcellence imaging software (Olympus). Experiments were performed at room temperature. F340/380 ratios were calculated for all regions of interest (ROI) after background subtraction. Stock solutions were diluted in HEPES buffered solution and manually added in a 1:10 ratio to the wells to reach a final concentration of 10 or 100 µM ATP (Tocris), 1 or 10 µM icilin (Tocris) and 1 or 10 µM capsaicin (Hello Bio). An F340/380 increase of >20 % of baseline was considered as response. Calcium imaging was performed after maturation day 50.

Clinical study

The observation CICARO cohort study was approved by the ethics committee of Charité – Universitätsmedizin Berlin (EA4/069/14, Berlin, Germany) and registered prior to recruitment at ClinicalTrials.gov (NCT02753036). Patients were screened in weekly interdisciplinary tumor board meetings and eligible patients scheduled for baseline visit V1, where written informed consent was obtained from participants prior to inclusion in the study. Patients had to fulfill the following inclusion criteria: 1) 18 to 70 years of age, 2) Karnofsky Index ≥70 %, 3) ≥8 years of school education. Patients with A) prior neurotoxic chemotherapy, B) former or current alcohol or drug abuse, C) mild cognitive impairment or dementia, D) post-surgery delirium, E) major depression or F) anemia <8 g/dl were excluded.

A total of n=72 patients were recruited to either one of three cohorts: otherwise healthy females, who underwent minor gynecological laparoscopic surgery due to benign tumors (‘healthy’ control), female breast cancer patients with anti-hormonal treatment but without chemotherapy (tumor ‘control’) and female ovarian and breast cancer patients treated with paclitaxel ± carboplatin (‘chemo’) and tested from 01/2016 (first patient in) to 09/2020 (last patient out). N=10 patients were lost to follow-up at V2

(withdrawal of consent, could not be reached, death) and subsequently excluded from the final analysis. Figure 2 summarizes the trial flow.

Neurological and electrophysiological examination

A neurological examination was conducted at study visits V1 (baseline, before chemotherapy) and 28 (range 14 to 45) weeks later (V2, after chemotherapy). Vibration sensitivity was measured with a commercial Riedel-Seyfert tuning fork and values of ≤6/8 were regarded as reduced. Muscle strength was documented according to the medical research council (MRC) scale. Reflex status was rated as normal, reduced or absent. Motor conduction velocity (MCV) was measured in one leg by a supramaximal stimulus of the common and deep peroneal nerve proximal of the ankle joint (S1) and under the head of the fibula (S2) by recording the corresponding compound motor action potentials (CMAP) with surface electrodes positioned over the M. extensor digitorum brevis using an Evidence ENG/EMG device (Schreiber & Tholen, Germany). Serial electric stimuli (at least 20) were applied to the lower calf and the sensory nerve action potential (SNAP) and sensory conduction velocity (SCV) of the sural nerve measured over the skin inferior to the lateral malleolus with surface electrodes. CIPN development was graded according to the Total Neuropathy Score reduced (13).

Neurofilament measurements

NFL and pNFH were measured by single molecule array (SIMOA) technology (Quanterix, Billerica, MA) in supernatants collected from iPSC-DSN cultures (Labor Berlin GmbH, Germany) and in patient sera (Naturwissenschaftliches und Medizinisches Institut, Germany) by operators blinded to clinical data.

Statistics

Patients lost to follow-up (n=10) were excluded from analysis. Final analysis was done with data from n=6 healthy controls, n=25 tumor controls and n=31 chemotherapy patients. Missing data was not imputed. Prism v9 (Graphpad Software, San Diego, CA)

and Stata v16 (StataCorp LLC, College Station, TX) were used for statistical analysis and data visualization. Gaussian distribution was checked with Shapiro-Wilk normality test before statistical analysis. Normally distributed data were analyzed using unpaired t-tests (2 group comparisons), whereas not normally distributed data were analyzed with Mann-Whitney-U test (2 group comparison) or Kruskal-Wallis test with Dunn's method for post hoc adjustment for multiple comparisons. Cell viability/cytotoxicity experiments with iPSC-DSN were replicated at least 3 times with at least 4 technical replicates. N=3 independent iPSC donor cell lines were used (for more detailed description on the replicates and the iPSC cell lines used for the respective experiments, please refer to the in-vitro part above). In-vitro NFL experiments were replicated at least 3 times, with 4 technical replicates pooled for each condition. Data is reported as median ± SD and displayed as median ± SD (in vitro data) or median with interquartile ranges (clinical data), unless otherwise stated in the figure legends. Non-linear regression analysis was performed to obtain dose-response curves (log-inhibitor vs. response, three parameters). Linear regression was performed with Pearson correlation (cell culture data, normally distributed) and with Spearman correlation (clinical data, no normal distribution) between 2 variables. Binary multiple logistic regression analysis was used to calculate predicted probabilities of CIPN development. Data is available on Mendeley Data (Huehnchen, Petra (2022), "Neurofilament proteins as potential biomarker in chemotherapy-induced polyneuropathy", Mendeley Data, V1, doi: 10.17632/w7w3myjpgc.1).

Supplemental Figures

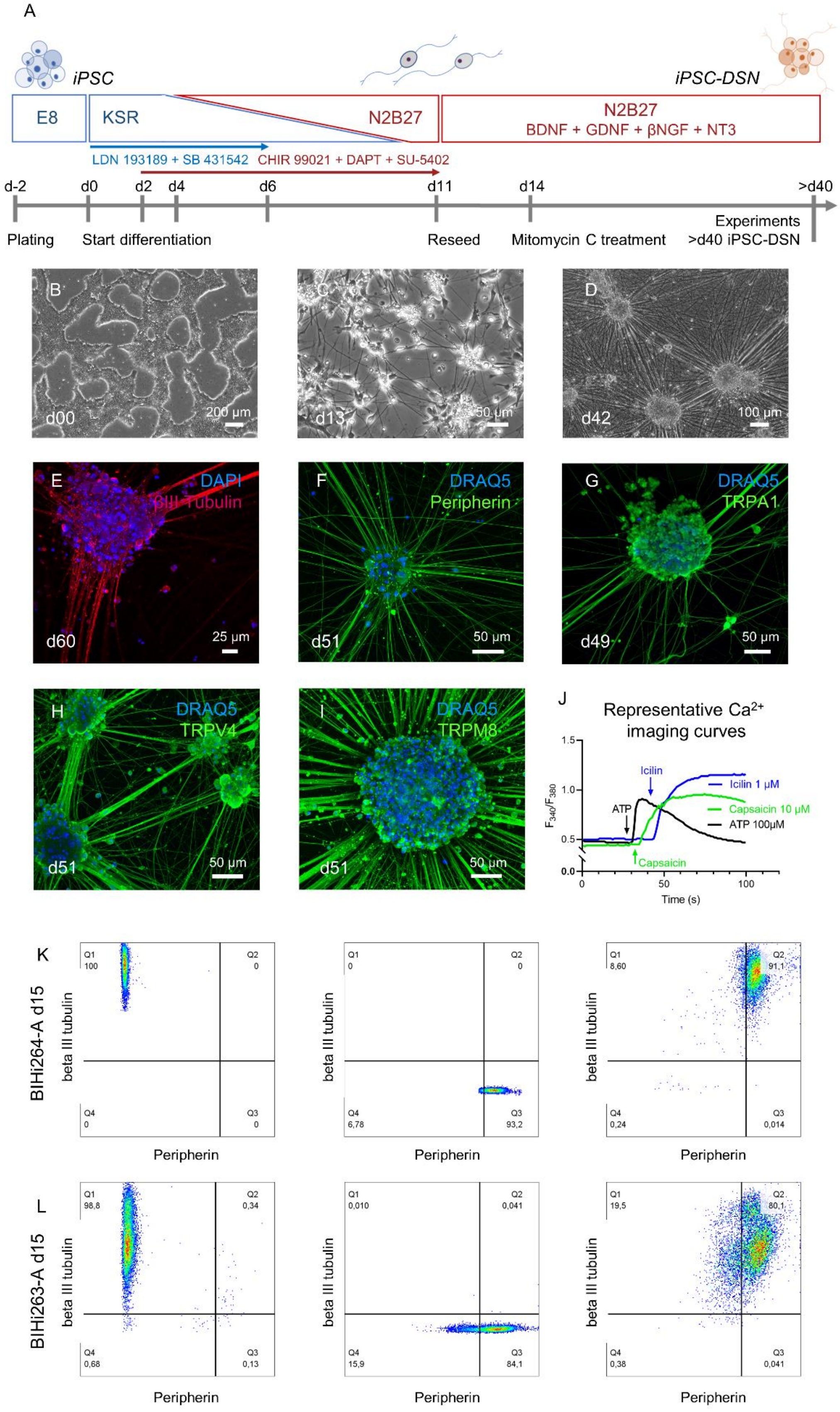

Supplemental Figure S1: Differentiation and characterization of hiPSC-DSN. (A) Schematic overview of differentiation protocol of hiPSC-DSN (adapted from (14)): Cells were cultured in E8 media until differentiation was started with the *2i* LDN19318 and SB431542 (SMAD inhibition), followed by the activation of WNT (CHIR99021), Notch inhibition (DAPT) and inhibition of VEGF/FGF/PDGF by SU5402. On d11, cells were reseeded and matured for at least 30 more days (*maturation phase*). (B-D) Representative phase contrast images of the differentiation phase: (B) from stem cell colonies to (C) bipolar-like neurons to (D) morphologically connected ganglia-like structures of hiPSC-DSN >40d. (E) Typical nervous system markers such as beta III tubulin and (F) peripherin as well as (G) the transient receptor potential cation channel subfamily A member 1 (TRPA1), (H) the subfamily V member 4 (TRPV4) and (I) the subfamily M member 8 (TRPM8) were expressed in hiPSC-DSN. (J) Human iPSC-DSN showed functional responses in calcium imaging experiments to inflammatory stimuli (ATP, 100 µM), the TRPV1 agonist capsaicin (1-10 µM) and the TRPM8 agonist icilin (1-10 µM). (K-L) Purity of iPSC-DSN cell lines BIHi264-A and BIHi263-A was investigated using fluorescence-activated cell sorting (FACS), confirming that *early* d15 iPSC-DSN already expressed on average 99.4 ± 0.9 % beta III tubulin and 88.7 ± 6.4 % peripherin while 85.6 ± 7.8 % of iPSC-DSN expressed both markers.

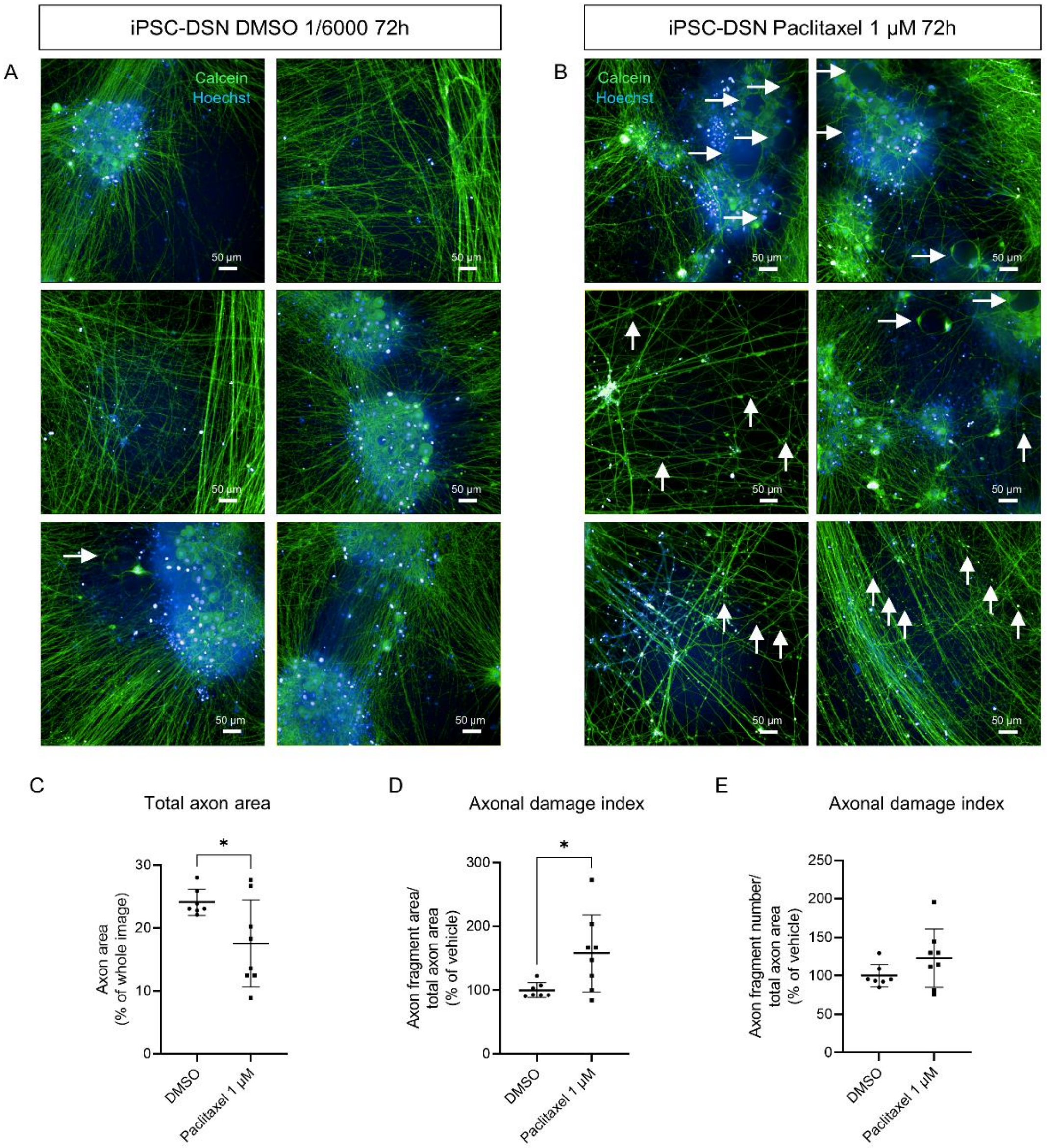

Supplemental Figure S2: Morphological changes and axonal damage of hiPSC-DSN upon 72h-treatment with paclitaxel. (A-B) In comparison to vehicle (DMSO), 72h treatment with paclitaxel at 1 µM leads to axonal blebbing (B, vertical arrow) in living cells and apoptotic cells (B, horizontal arrow). Few apoptotic cells were also observed in vehicle (A, lower left panel), whereas axonal degeneration was not (scale bar 50 µm). (C) In comparison to DMSO treated neurons, paclitaxel incubation at 1 µM for 72h was associated with axonal thinning as less surface was covered by axons, (D) an increased axonal damage index as a larger area was covered with axonal fragments in relation to total axon area and (E) a tendency to show more fragments

per total axon area in paclitaxel treated hiPSC-DSN (p=0.16). *Statistical analysis:* (C-E) n=7 vs. n=8 images, unpaired t-tests.

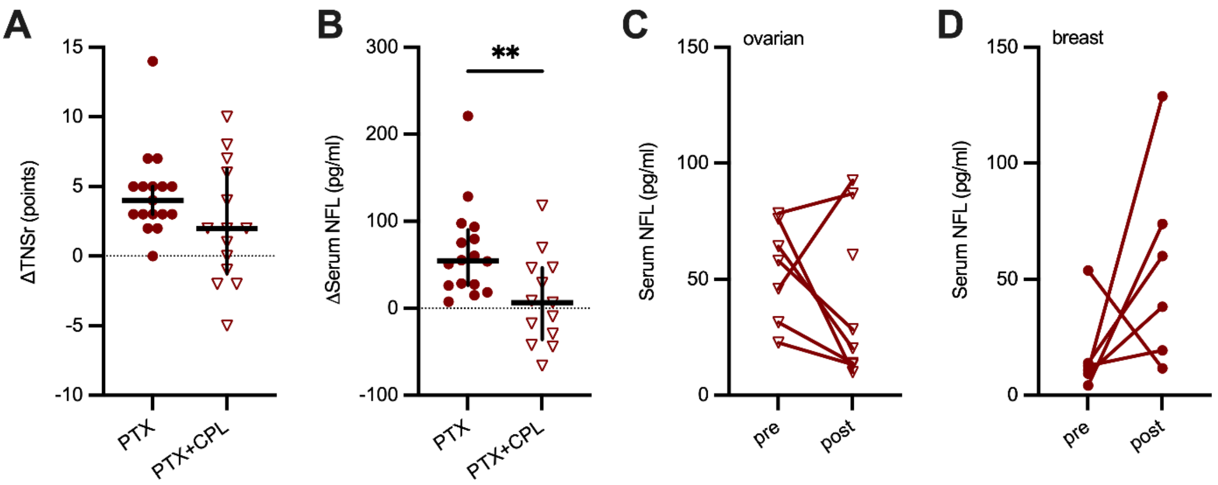

Supplemental Figure S3: CIPN development and changes in serum NFL (NFL_S) in patients with paclitaxel/ carboplatin combination therapy. (A) Patients with a paclitaxel/ carboplatin combination therapy (PTX+CPL) did not have higher TNSr values than patients who were treated with PTX alone. (B) Changes in NFL_S concentrations in patients with PTX+CPL were lower compared to patients with single PTX therapy. However, results are likely skewed as (C) ovarian cancer patients, who are routinely treated with PTX+CPL, had higher baseline NFL_S concentrations than (F) breast cancer patients. *Statistical analysis:* (A-B) Kruskal-Wallis test. *Subjects:* (A-B) n=17 (PTX), n=14 (PTX+CPL); (C-D) n=8 (ovarian), n=6 (breast). *p<0.05, **p<0.01, ***p<0.001, ****p<0.0001, ns not significant.

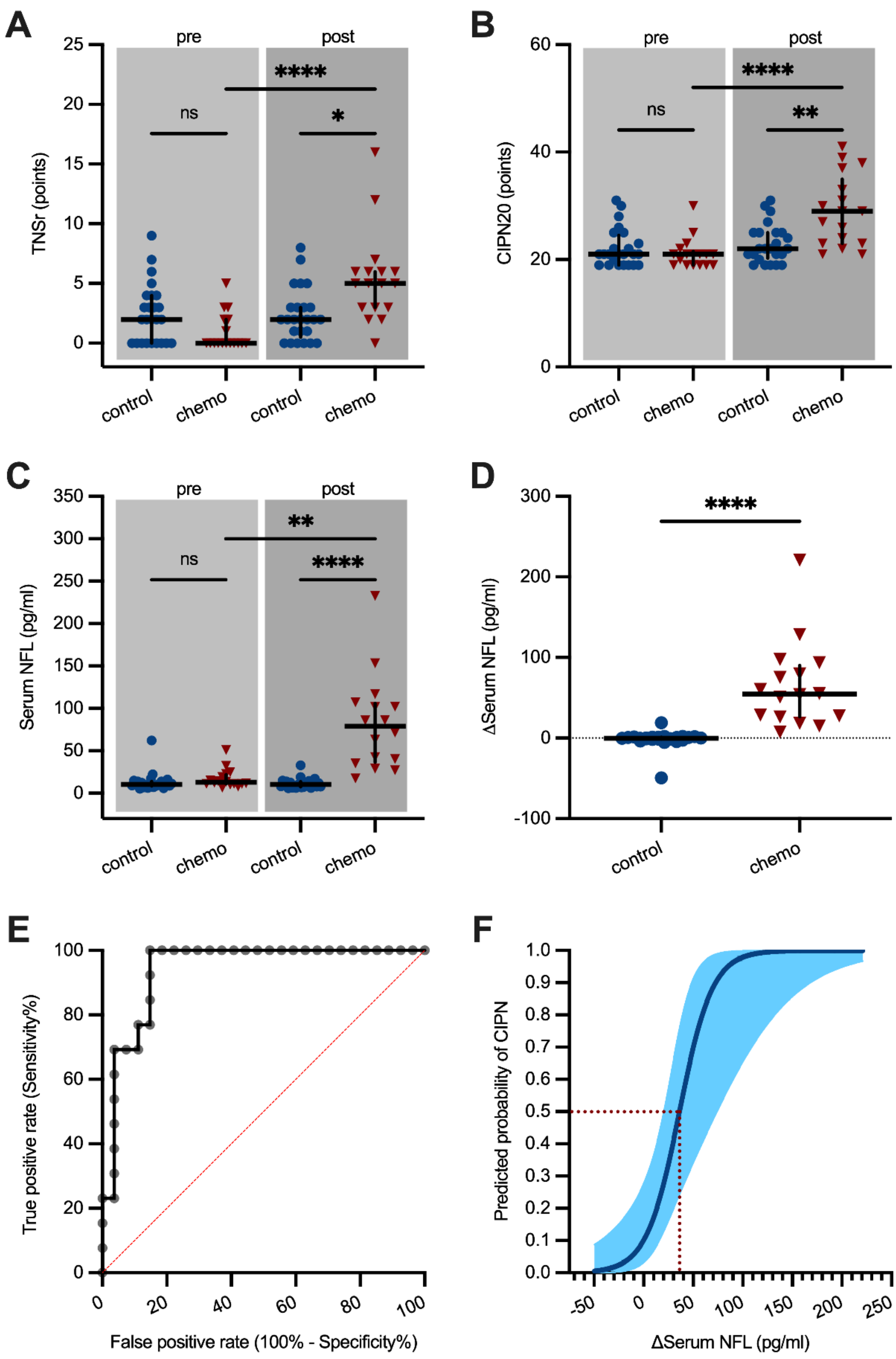

Supplemental Figure S4: CIPN development and diagnostic properties of NFL_s in patients with paclitaxel monotherapy. (A) The TNSr increased in patients, who only received paclitaxel monotherapy compared to breast cancer control patients similarly as in the entire cohort. (B) The same results were observed in patient-reported

data (CIPN20 questionnaire). (C+D) NFL_S values were very comparable across both groups at baseline and significantly increased in patients after paclitaxel monotherapy. (E) Receiver operating characteristics analysis revealed that the sensitivity of ΔNFL_S was better when only patients with paclitaxel monotherapy were included in the analysis. (F) The threshold of a predicted probability to have CIPN of >0.5 was similar in patients with paclitaxel monotherapy compared to the analysis of the entire cohort (area filling indicates 95 % CI). *Statistical analysis:* (A-C) Kruskal-Wallis test, (D) Mann-Whitney-U test, (E) ROC, (F) logistic regression analysis. *Subjects:* n=25 (control), n=17 (chemo). *p<0.05, **p<0.01, ***p<0.001, ****p<0.0001, ns not significant.

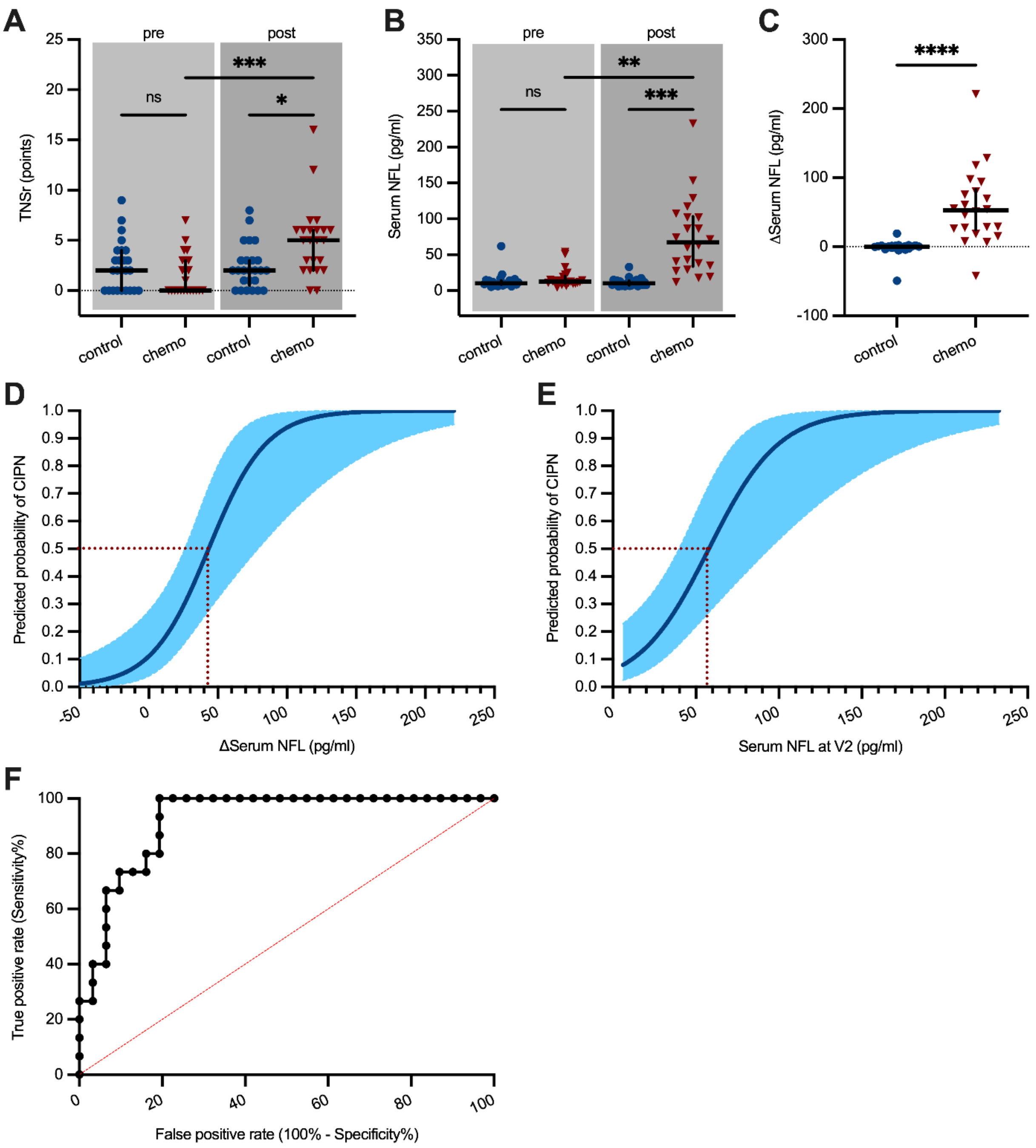

Supplemental Figure S5: CIPN development and diagnostic properties of NFLs in breast cancer patients. (A) We observed similar results in TNSr development when only breast cancer patients were included in the analysis. (B+C) NFLs values equally strong increased in the breast cancer cohort compared to the entire cohort. (D) Logistic regression analysis revealed that the threshold for ΔNFLs for a predicted probability of >0.5 to have CIPN was slightly higher in the breast cancer cohort compared to the analysis of all patients (area filling indicates 95 % CI). (E) Similar results were observed

for NFL_s levels at V2 (area filling indicates 95 % CI). (F) Sensitivity of ΔNFL_s increased to 100 % while specificity slightly decreased to 81 % in a ROC analysis including only breast cancer patients. *Statistical analysis:* (A+B) Kruskal-Wallis test, (C) Mann-Whitney-U test, (D+E) logistic regression analysis, (F) ROC. *Subjects:* n=25 (control), n=23 (chemo). *p<0.05, **p<0.01, ***p<0.001, ****p<0.0001, ns not significant.

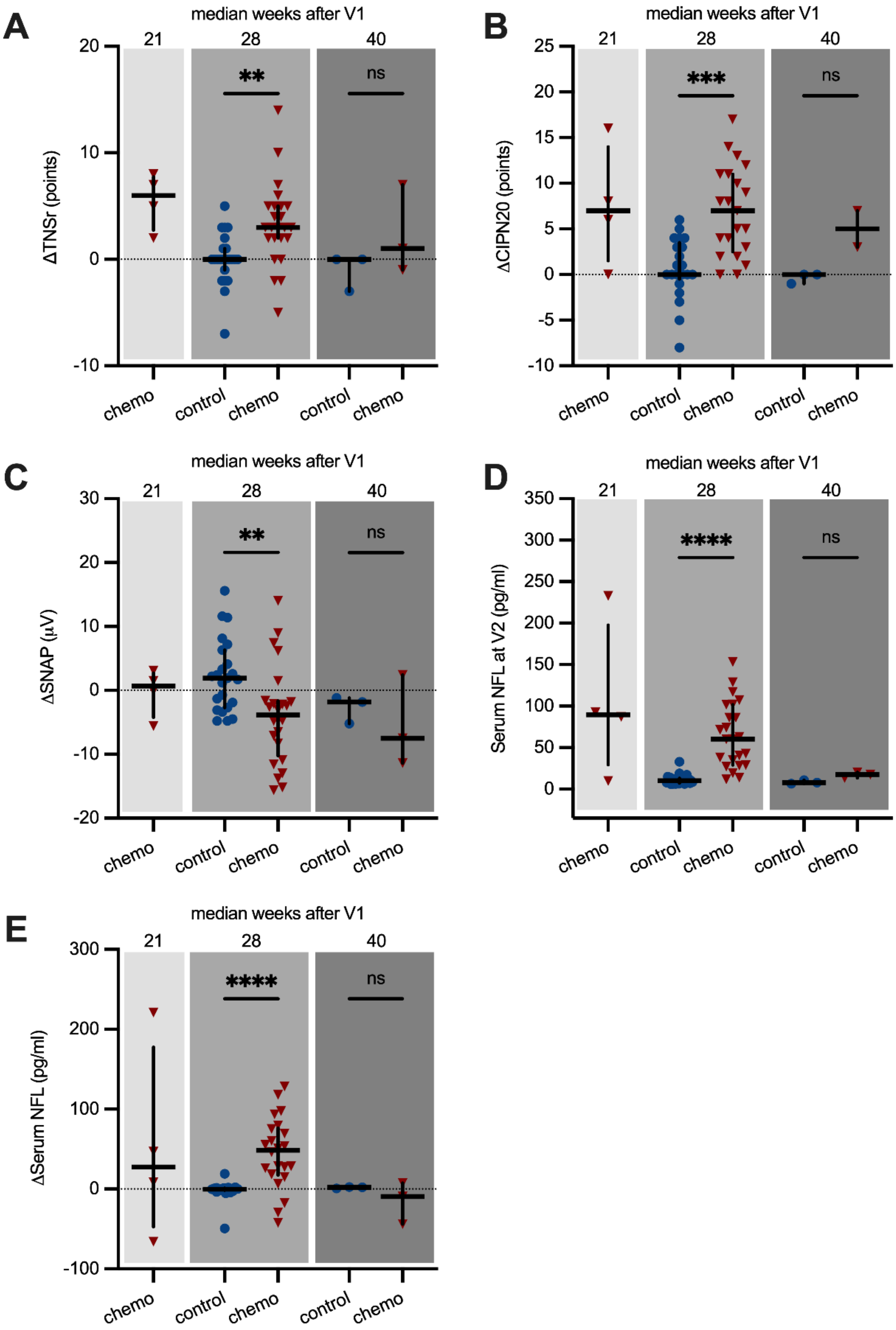

Supplemental Figure S6: CIPN characteristics depending on V2 time point. (A) Increase in TNSr was highest at the earliest V2 time point and afterwards steadily declined almost reaching normal values again 40 weeks after V1. (B) Similar findings were observed in patient-reported outcome measures of CIPN (CIPN20

questionnaire). (C) The impact of neurotoxic chemotherapy on the structural integrity of the sural nerve as measure of neurodegeneration could be detected at the earliest after 28 weeks and persisted until 40 weeks after V1. (D) NFL_s values at V2 were highest at 21 weeks and then steadily declined reaching similar levels as controls at 40 weeks after V1. (E) Similar results could be observed for the increase of NFL_s compared to baseline. *Statistical analysis:* (A-E) Kruskal-Wallis test. *Subjects:* 21 weeks: n=4 (chemo); 28 weeks: n=23 (control), n=24 (chemo); 40 weeks: n=3 (control), n=3 (chemo). *p<0.05, **p<0.01, ***p<0.001, ****p<0.0001, ns not significant.

Supplemental Tables

Supplemental Table T1: results from FACS-analysis of two iPSC-DSN lines (BIHi263-A and BIHi264-A) regarding the expression of beta III tubulin and peripherin.

	beta-III tubulin positive [%]	Peripherin positive [%]	Combined positive [%]
**BIHi-264a, d15, thawed**	100	93.2	91.1
**BIHi-263a, d15, thawed**	98.8	84.1	80.1
**Average**	99.4	88.65	85.6
**Standard Deviation**	0.85	6.43	7.78

References

1. Hennig AF, Rössler U, Boiti F, von der Hagen M, Gossen M, Kornak U, et al. Generation of a human induced pluripotent stem cell line (BIHi002-A) from a patient with CLCN7-related infantile malignant autosomal recessive osteopetrosis. *Stem Cell Res.* 2019;35:101367.
2. Fusaki N, Ban H, Nishiyama A, Saeki K, and Hasegawa M. Efficient induction of transgene-free human pluripotent stem cells using a vector based on Sendai virus, an RNA virus that does not integrate into the host genome. *Proc Jpn Acad Ser B Phys Biol Sci.* 2009;85(8):348-62.
3. Cernoch J, Fisch T, Fischer I, Fischer K, Iwanska A, Kruger N, et al. Generation of 20 human induced pluripotent stem cell lines from patients with focal segmental glomerulosclerosis (FSGS). *Stem Cell Res.* 2021;54:102406.
4. Hennig AF, Rössler U, Boiti F, Von Der Hagen M, Gossen M, Kornak U, et al. Generation of a human induced pluripotent stem cell line (BIHi002-A) from a patient with CLCN7-related infantile malignant autosomal recessive osteopetrosis. *Stem Cell Research.* 2019;35:101367.
5. Schwartzentruber J, Foskolou S, Kilpinen H, Rodrigues J, Alasoo K, Knights AJ, et al. Molecular and functional variation in iPSC-derived sensory neurons. *Nat Genet.* 2018;50(1):54-61.
6. Gendron TF, Badi MK, Heckman MG, Jansen-West KR, Vilanilam GK, Johnson PW, et al. Plasma neurofilament light predicts mortality in patients with stroke. *Science translational medicine.* 2020;12(569).
7. Huehnchen P, Boehmerle W, Springer A, Freyer D, and Endres M. A novel preventive therapy for paclitaxel-induced cognitive deficits: preclinical evidence from C57BL/6 mice. *Transl Psychiatry.* 2017;7(8):e1185.
8. Sasaki Y, Vohra BPS, Lund FE, and Milbrandt J. Nicotinamide Mononucleotide Adenylyl Transferase-Mediated Axonal Protection Requires Enzymatic Activity But Not Increased Levels of Neuronal Nicotinamide Adenine Dinucleotide. *Journal of Neuroscience.* 2009;29(17):5525-35.
9. Schinke C, Fernandez Vallone V, Ivanov A, Peng Y, Körtvelyessy P, Nolte L, et al. Modeling chemotherapy induced neurotoxicity with human induced pluripotent stem cell (iPSC) -derived sensory neurons. *Neurobiology of disease.* 2021;155:105391.
10. Schinke C, Fernandez Vallone V, Ivanov A, Peng Y, Körtvelyessy P, Nolte L, et al. Dataset for: Modeling chemotherapy induced neurotoxicity with human induced pluripotent stem cell (iPSC)-derived sensory neurons. *Data Brief.* 2021;38:107320.
11. Stacey P, Wassermann AM, Kammonen L, Impey E, Wilbrey A, and Cawkill D. Plate-Based Phenotypic Screening for Pain Using Human iPSC-Derived Sensory Neurons. *SLAS DISCOVERY: Advancing Life Sciences R&D.* 2018;23(6):585-96.
12. Von Der Ahe D, Huehnchen P, Balkaya M, Peruzzaro S, Endres M, and Boehmerle W. Suramin-Induced Neurotoxicity: Preclinical Models and Neuroprotective Strategies. *Molecules (Basel, Switzerland).* 2018;23(2):346.
13. Cavaletti G, Frigeni B, Lanzani F, Piatti M, Rota S, Briani C, et al. The Total Neuropathy Score as an assessment tool for grading the course of chemotherapy-induced peripheral neurotoxicity: comparison with the National Cancer Institute-Common Toxicity Scale. *J Peripher Nerv Syst.* 2007;12(3):210-5.
14. Schinke C, Vallone VF, Ivanov A, Peng Y, Körtvelyessy P, Nolte L, et al. Modeling chemotherapy induced neurotoxicity with human induced pluripotent stem cell (iPSC) -derived sensory neurons. *Neurobiol Dis.* 2021:105391.
